# Synergistic γ‐In_2_Se_3_@rGO Nanocomposites with Beneficial Crystal Transformation Behavior for High‐Performance Sodium‐Ion Batteries

**DOI:** 10.1002/advs.202303108

**Published:** 2023-08-04

**Authors:** Yun Zhao, Haoyue Zhang, Yong Li, Canliang Ma, Wenjuan Tian, Xingguo Qi, Gaoyi Han, Zongping Shao

**Affiliations:** ^1^ Institute of Molecular Science Key Laboratory of Materials for Energy Conversion and Storage of Shanxi Province Key Laboratory of Chemical Biology and Molecular Engineering of Education Ministry Shanxi University Taiyuan 030006 P. R. China; ^2^ Shanxi‐Zheda Institute of Advanced Materials and Chemical Engineering Taiyuan 030006 P. R. China; ^3^ Research Center for Fine Chemicals Engineering Shanxi University Taiyuan 030006 P. R. China; ^4^ Shanxi Huana Carbon Energy Technology Co. Ltd. Taiyuan 030006 P. R. China; ^5^ WA School of Mines: Minerals Energy and Chemical Engineering (WASM‐MECE) Curtin University Perth WA 6102 Australia

**Keywords:** crystal transformation, In_2_Se_3_, reduced graphene oxide, sodium‐ion batteries, synergistic effect

## Abstract

Crystal transformation of metal compound cathodes during charge/discharge processes in alkali metal‐ion batteries usually generates profound impact on structural stability and electrochemical performance, while the theme in anode materials, which always occurs and completes during the first redox cycle, is rarely explored probably due to the fast transformation dynamics. Herein, for the first time, a unique crystal transformation behavior with slow dynamics in anode of sodium‐ion batteries (SIBs) is reported, which further promotes electrochemical performance. Specifically, irreversible γ → β crystal transformation of In_2_Se_3_ is observed, induced by the persistent size degradation of In_2_Se_3_ particles during repeated sodiation/desodiation, supported by a series of ex situ characterizations, such as HRTEM, XRD, and XPS of γ‐In_2_Se_3_/reduced graphene oxide (γ‐In_2_Se_3_@rGO) nanocomposite. The hybrid electrode shows ultrahigh long‐term cycling stability (378 mA h g^−1^ at 1.0 A g^−1^ after 1000 cycles) and excellent rate capability (272 mA h g^−1^ at 20.0 A g^−1^). Full battery with Na_3_V_2_(PO_4_)_3_ cathode also manifests superior performance, promising β‐In_2_Se_3_ dominated electrode materials in high‐power and long‐life SIBs. The first‐principle calculations suggest the crystal transformation enhances electric conductivity of β‐In_2_Se_3_ and facilitates its accessibility to sodium. In combination with the synergistic effect between rGO matrix, substantially enhanced electrochemical performance is realized.

## Graphical abstract

A beneficial crystal transformation in metal compound anode materials is systematically explored for sodium‐ion batteries (SIBs) for the first time. A unique γ → β phase transformation of In_2_Se_3_, which is triggered by the persistent size degradation of active nanoparticles, occurred during the repeated sodiation/desodiation process, contributing to substantially enhanced cycling stability and rate capability for γ‐In_2_Se_3_@rGO electrode materials.

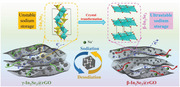

## 1 Introduction

The next‐generation electric power system based on renewable energies is one of the fundamental solutions to alleviate global fossil energy exhaustion and the issues of excessive carbon emissions. Because of the intermittent nature and scattering region dispersion of the renewable power sources, such as solar, wind, and tide energies, large‐scale energy storage devices are indispensable to guarantee the grid‐connection stability and the flexibility to terminal consumers.^[^ ^1^, ^2^ ^]^ Amidst of the promising large‐scale energy storage technologies, sodium‐ion batteries (SIBs) stand out by the virtues of high energy density, high operation voltage, long cycling life, and low cost. Moreover, the similar working principle to that in lithium‐ion batteries (LIBs) and the abundant sodium resources make SIBs be highly competitive for commercial applications.^[^ ^3^, ^4^, ^5^, ^6^, ^7^ ^]^ Nevertheless, the practical applications of SIBs are still frustrated by the deficiency of commercially suitable electrode materials. Especially, the commonly used artificial/natural graphites as commercial anode materials of LIBs are failed in SIBs due to the thermodynamic instability of the sodium‐graphite intercalation compounds.^[^ ^8^, ^9^ ^]^

For promoting the commercial applications of SIBs, tremendous efforts have been made to exploit high‐performance anode materials, such as hard carbon, soft carbon, and metal chalcogenides.^[^ ^10^, ^11^, ^12^ ^]^ Among them, metal selenides (MSs) with conversion or/and alloy type sodium‐storage mechanisms are receiving ever‐increasing research interests owing to their high theoretical specific capacity, safe working voltage platform, and environmental friendly nature.^[^ ^13^ ^]^ Moreover, the weaker covalent bonds and narrower band gaps of MSs than that for the corresponding metal oxides and sulfides result in easier sodiation/desodiation reaction kinetics and better electric conductivity. Therefore, many successful cases, such as CoSe_2_,^[^ ^14^ ^]^ MoSe_2_,^[^ ^15^ ^]^ SnSe,^[^ ^16^ ^]^ and Sb_2_Se_3_,^[^ ^17^ ^]^ have been demonstrated to manifest favorable sodium‐storage performance. Nevertheless, like other metal‐based compounds with high capacity (e.g., sulfides and phosphides), the intrinsic low electron/ion conductivity and enormous volume fluctuation upon conversion/alloy reactions always remain to be challenging for desirable performance. For promoting electrochemical performance, the rational nanostructure design and the combination with high‐conductive carbon materials (carbon coatings or carbon matrices) have been widely practiced as the most effective strategies.^[^ ^18^, ^19^, ^20^, ^21^, ^22^ ^]^ Besides, other potential strategies that may boost the electrode capacity and cycling stability are also intensively searched after.

It is well known that crystal phase transformation behaviors of metal compound electrode materials during charge/discharge processes exert a profound influence on structural stability and electrochemical performance for alkali‐metal ion batteries.^[^ ^23^, ^24^ ^]^ These phenomena always occur in cathode materials with the induction of extraction of alkali‐metal ions at high charge voltage. For example, during the delithiation process of LiCoO_2_ cathodes, the migration of cobalt ions into lithium layers leads to the phase transition of layered structure to spinel and rock‐salt structures.^[^ ^25^ ^]^ Tremendous interests have been drawn to the researches of phase evolutions in sodium‐based transition metal layered oxide cathodes (Na_x_TMO_2_) in recent years. The dominating P2‐type and O3‐type phase structures for Na_x_TMO_2_ are transformed to O2 and P3 separately at the deeply charged states (above 4.2 V) due to the gliding of oxygen layers, which unfortunately always result in the lattice mismatch and dislocations.^[^ ^26^, ^27^ ^]^ As a consequence, the irreversible phase transitions give rise to a series of detrimental effects, including the deteriorated electrode structures and degraded cycling stability. Therefore, normally such crystal transformations should be avoided or suppressed through limiting the operation voltage window and cation doping in transition metal sites.^[^ ^27^ ^]^

Surprisingly, the systematic crystal phase transformation investigations were rarely carried out in the literatures relating to metal compound anode materials, especially for SIBs, which could be resulted from the too fast phase transformation dynamics during electrochemical redox processes according to the reported cases currently. For example, a rapid crystal transformation of MnS from α to β phase was observed during the first discharge/charge cycle in LIBs, while the analogous behavior did not appear in the case of SIBs.^[^ ^28^ ^]^ And recently, a fast phase transformation from Cu_2_Se to Cu_2‐x_Se was also discovered in the sodiation/desodiation process of the first cycle, but the related intensive studies were scarce.^[^ ^29^, ^30^ ^]^ We speculate these metal compounds with rich crystal phases may have a higher probability for the occurrence of crystal transformation upon redox reactions. Similar to MnS, In_2_Se_3_ is a typical polymorphic chalcogenide that has various of phase structures such as α, β, γ, δ, and к.^[^ ^31^ ^]^ As an important member in MSs, In_2_Se_3_ is a typical III‐VI chalcogenide semiconductor with a 2D layered structure in most of cases. Benefitting from the featured bandgap of 1.3–2.0 eV, it has become hotspots in the fields of solar cells,^[^ ^32^ ^]^ photocatalysis,^[^ ^33^, ^34^ ^]^ phase change memory,^[^ ^35^ ^]^ and gas sensors.^[^ ^36^ ^]^ For the electrochemical energy storage applications, In_2_Se_3_ also exhibits great potentials due to its competitive reversible capacity and high volumetric energy density. However, up to now, there are still few reports about the studies relating to In_2_Se_3_ based electrode materials for SIBs.^[^ ^37^, ^38^ ^]^

In this study, for the first time, we reported a unique and beneficial crystal transformation phenomenon in SIBs by employing In_2_Se_3_ as the anode material. Considering the low intrinsic conductivity, it was coupled with reduced graphene oxide (rGO) through a facile wet chemistry method. In particular, one kind of γ‐In_2_Se_3_@rGO nanocomposite was developed as a superb prototype. The repeated sodiation/desodiation induced crystal transformation was systematically investigated by various material characterizations, especially the ex‐situ means such as HRTEM observation and XRD/XPS analyses. The electrochemical behavior and kinetics investigation demonstrated that the γ → β crystal transition of In_2_Se_3_ contributed to the ultra‐stable cycling ability and excellent rate performance. Such beneficial crystal transformation then provides another strategy for the development of competitive anodes for SIBs.

## 2 Results and Discussion

### 2.1 Synthesis of the γ‐In_2_Se_3_@rGO Nanocomposites

**Figure 1:**
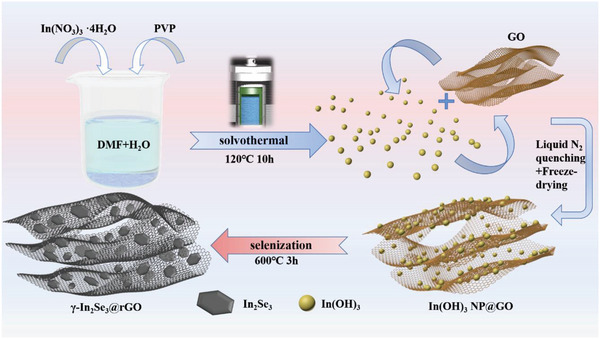
Schematic illustration for the preparation process of the γ‐In_2_Se_3_@rGO nanocomposites.

The γ‐In_2_Se_3_@rGO nanocomposites were prepared by first liquid‐phase mixing of In(OH)_3_ nanoparticles (NPs) and GO and then conducting a gas phase selenization treatment, as schematically illustrated in **Figure** **1**. In(OH)_3_ NPs were synthesized via a solvothermal route with DMF/H_2_O mixture as the solvent, which provided a weak basic medium based on the hydrolysis mechanism:^[^ ^39^ ^]^ $\mathrm{HOCN}{\left({\mathrm{CH}}_{3}\right)}_{2}+H_{2}O\to \mathrm{HOCNH}{\left({\mathrm{CH}}_{3}\right)}_{2}^{+}+{\mathrm{OH}}^{-}$, to conduct the nucleation and crystal growth of the In(OH)_3_ species. Meanwhile, PVP was added in to play the roles of surface stabilizer and particle dispersant,^[^ ^40^ ^]^ contributing to the small dimension of the resulting In(OH)_3_ NPs. In(OH)_3_@GO nanocomposite was then obtained by mixing In(OH)_3_ NPs and GO in distilled water, then quenching in liquid N_2_ and finally conducting freeze‐drying. As shown in Figure S1 (Supporting Information), the In(OH)_3_ NPs were evenly dispersed on the surface of GO, and the dimension of most of the particles was less than 50 nm. The high purity of these In(OH)_3_ NPs was verified by XRD analysis (Figure S2, Supporting Information). Subsequently, the In(OH)_3_@GO nanocomposite was mixed with selenium powder and heat‐treated in an Ar atmosphere to realize the transformation from In(OH)_3_ phase to γ‐In_2_Se_3_ phase. The possible selenization mechanism is as: 1) 2In(OH)_3_ → In_2_O_3_ + 3H_2_O; 2) 2In_2_O_3_  + 9Se  →  2In_2_Se_3_  +  3SeO_2_ since the two‐step reaction process between In_2_O_3_ and Se which produces In^0^ transient phase may not occur after monitoring the products at different selenization temperature (Figure S3, Supporting Information). The selenization procedure meanwhile accompanied the reduction of GO, leading to the formation of the γ‐In_2_Se_3_@rGO nanocomposites.

**Figure 2:**
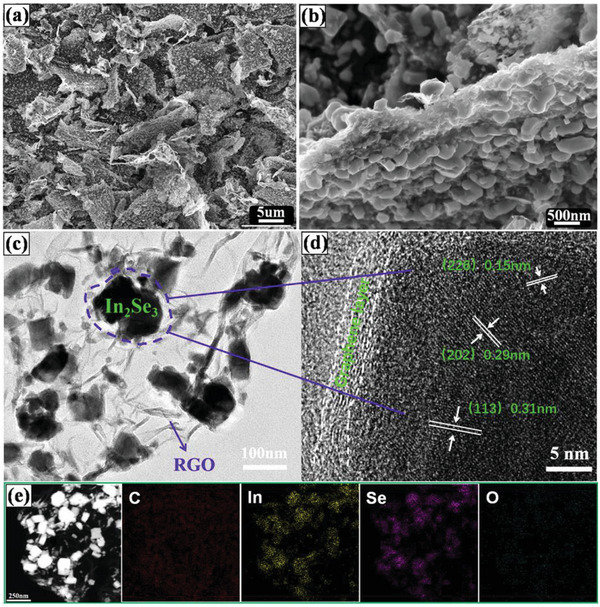
a,b) SEM images, c) TEM, and d) HRTEM images of the γ‐In_2_Se_3_@rGO‐0.06 nanocomposite. e) The HAADF‐STEM image and the corresponding EDX elemental mapping.

**Figure** **2** and Figure S4 (Supporting Information) present the morphology of the γ‐In_2_Se_3_@rGO nanocomposites with different loading amount of In_2_Se_3_. The selenization process resulted in an obvious size increase of the as‐obtained In_2_Se_3_ NPs. Even for the sample with the lowest In_2_Se_3_ loading amount (γ‐In_2_Se_3_@rGO‐0.2 nanocomposite), the particle size prominently increased from <50 nm for the In(OH)_3_ precursor to several hundreds of nanometers for these In_2_Se_3_ NPs, which could be a result from the sintering effect and high reactivity of the adjacent In(OH)_3_ NPs. A shape transformation of the cube‐like particles into hexangular sheet‐stacked morphology during the selenization process was also observed, which can be explained by the hexagonal crystalline nature of In_2_Se_3._ In the γ‐In_2_Se_3_@rGO‐0.2 nanocomposite, according to Figure S4a,b (Supporting Information), the In_2_Se_3_ NPs were homogeneously anchored on the wrinkled surface of rGO. Some tiny In_2_Se_3_ NPs were also detected. The high monodispersibility of rGO was a result from the cold‐quenching and the following freeze‐drying treatment.^[^ ^41^ ^]^ With the increase of In(OH)_3_ feedstock, the loading density of In_2_Se_3_ on the rGO matrix increased. For the γ‐In_2_Se_3_@rGO‐0.06 nanocomposite (Figure 2a,b), the In_2_Se_3_ NPs still maintained a uniform dispersion without obvious size increase, while the further augmented In(OH)_3_ addition proportion (the γ‐In_2_Se_3_@rGO‐0.01 nanocomposite) gave rise to poor dispersibility and serious sintering of the resulting In_2_Se_3_ NPs, as shown in Figure S4c,d (Supporting Information). Furthermore, the bare In_2_Se_3_ sample derived from the direct selenization of In(OH)_3_ NPs demonstrated a similar sintering behavior to that in the γ‐In_2_Se_3_@rGO‐0.01 nanocomposite and an agglomerated morphology (Figure S4e,f, Supporting Information ), indicating rGO played important roles in the dispersibility of In_2_Se_3_ NPs. In addition, the electric conductivity of In_2_Se_3_ was prominently improved after the combination with rGO. As shown in Table S1 (Supporting Information), the electric conductivity steadily rises from 0.010 S cm^−1^ of bare In_2_Se_3_ to 0.071 S cm^−1^ of the γ‐In_2_Se_3_@rGO‐0.2 nanocomposite as increasing rGO content, further reflecting the favorable association state between In_2_Se_3_ NPs and rGO.

The weight proportions of In_2_Se_3_ in the γ‐In_2_Se_3_@rGO nanocomposites were determined by TG analyses. As shown in Figure S5 (Supporting Information), the weight loss below 200 °C was related to the adsorbed or crystal water, while the slight mass increase before 400 °C arose from the oxidation of In_2_Se_3_ to form In_2_O_3_ and SeO_2_. The subsequent stepwise weight loss was ascribed to the consumption of carbon and the sublimation of SeO_2_.^[^ ^42^ ^]^ The detailed chemical reaction equations are summarized below:

(1) $$2In_{2}{\mathrm{Se}}_{3}+9O_{2}\to 2In_{2}O_{3}+6\mathrm{Se}O_{2}↑\;$$

(2) $$C+O_{2}\to {\mathrm{CO}}_{2}↑$$

Based on the sequential thermal transition behaviors, the weight contents of In_2_Se_3_ in the samples can be calculated by the formula: $\frac{\mathrm{molar}\;\mathrm{mass}\;({\mathrm{In}}_{2}{\mathrm{Se}}_{3})}{\mathrm{molar}\;\mathrm{mass}\;({\mathrm{In}}_{2}O_{3})}\times \mathrm{weight}\;\mathrm{percentage}\;\left({\mathrm{In}}_{2}O_{3}\right)$. As a result, the percentages of In_2_Se_3_ in the γ‐In_2_Se_3_@rGO‐0.2, γ‐In_2_Se_3_@rGO‐0.06 and γ‐In_2_Se_3_@rGO‐0.01 nanocomposites were estimated to be 54.5 wt.%, 84.0 wt.%, and 96.6 wt.%, respectively. Correspondingly, the approximate proportions of rGO and PVP‐derived pyrolysis carbon can be calculated, as summarized in Table S2 (Supporting Information).

Considering both high loading amount and favorable dispersibility of In_2_Se_3_ in the γ‐In_2_Se_3_@rGO‐0.06 nanocomposite, its microstructure was further investigated by TEM and HRTEM. According to Figure 2c, the wrinkle‐rich surface characteristic and the thin lateral sheets of rGO were distinctly observed, which were resulted from the inclination to minimize surface energy during the cold‐quenching and solvent‐removal process. The wrinkled surface inhibited the restack and agglomeration of GO sheets. As a result, the monodispersed rGO was well retained after the heat treatment. It is also noted that the In_2_Se_3_ NPs were firmly anchored on or wrapped in rGO, which was benefited from the cold‐quenching by liquid N_2_.^[^ ^43^ ^]^ The compact association between the In_2_Se_3_ NPs and rGO was more clearly reflected in the HRTEM image (Figure 2d). In addition, the tiny crystals that expose different crystalline planes on surface were observed, implying the polycrystalline nature of the In_2_Se_3_ NPs. The determined lattice fringe distances of 0.15, 0.29, and 0.31 nm were indexed to (226), (202), and (113) planes for In_2_Se_3_ (JCPDS 40–1407). The high angle annular dark field scanning TEM (HAADF‐STEM) image and the corresponding EDX elemental mapping were shown in Figure 2e. The elemental dispersion of In and Se overlapped well, which concentrated in the white particle regions in the STEM image, intuitively proving the existence of pure In_2_Se_3_ particles on the surface of rGO. The detected O element was attributed to residual oxygen functional groups on the surface of rGO after heat treatment.^[^ ^44^ ^]^

**Figure 3:**
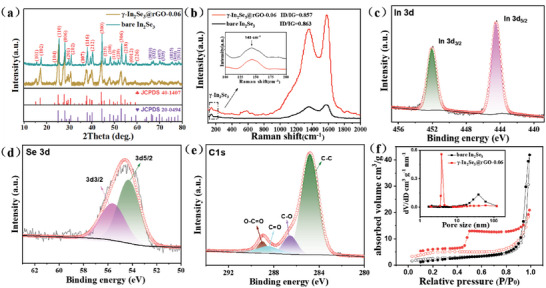
a) XRD patterns and b) Raman spectra of the γ‐In_2_Se_3_@rGO‐0.06 nanocomposite and bare In_2_Se_3_. The high‐resolution XPS spectra of In 3d c), Se 3d d), and C 1s e) for the γ‐In_2_Se_3_@rGO‐0.06 nanocomposite. f) N_2_ adsorption‐desorption isotherms of the γ‐In_2_Se_3_@rGO‐0.06 nanocomposite and bare In_2_Se_3_.

The crystalline phase compositions of the γ‐In_2_Se_3_@rGO nanocomposites were analyzed by XRD. It is seen from **Figure** **3**a and Figure S6 (Supporting Information) that the γ‐In_2_Se_3_@rGO nanocomposites and bare In_2_Se_3_ had roughly the same patterns, indicating the introduction of the rGO matrix did not affect the transformation of In_2_Se_3_ phase from In(OH)_3_. The slightly lower peak intensity and increased peak width would be attributed to the reduced particle size of In_2_Se_3_ and improved dispersity after the introduction of rGO. The diffraction peaks can be well indexed to mixed phases of γ‐In_2_Se_3_, which consisted of a major phase (JCPDS 40–1407, *a* = *b* = 7.13 Å, *c* = 19.38 Å), and a minor phase (JCPDS 20–0494, *a* = *b* = 7.10 Å, *c* = 19.31 Å). The γ‐In_2_Se_3_ had a hexagonal crystal structure with distorted wurtzite‐type atomic arrangement.^[^ ^45^ ^]^ The helical tetrahedral bonding characteristic in crystal cells is shown in Figure S7 (Supporting Information). Raman spectra were further conducted to reveal the texture of the carbon components (Figure 3b). For the γ‐In_2_Se_3_@rGO‐0.06 nanocomposite, two distinct peaks at 1590 cm^−1^ (G band) and 1360 cm^−1^ (D band) were related to the lattice vibration from sp^2^‐hybridized ordered carbon atoms and defect or disordered structures in carbon materials, respectively.^[^ ^46^ ^]^ The high intensity ratio of 0.86 between D band and G band (*I*_D_/*I*_G_) signifies the low restack degree of graphene layers and high surface or edge defects for facilitating the association with In_2_Se_3_ NPs. The two characteristic bands were also detected in the spectrum of bare In_2_Se_3_, which were originated from the PVP‐derived pyrolysis carbon. In addition, a discernible peak appearing at ≈143 cm^−1^ was attributed to the A_1g_^1^ phonon vibration mode of γ‐In_2_Se_3_.^[^ ^47^ ^]^

The surface chemical composition and bonding states of elements were measured by XPS. As shown in Figure S8 (Supporting Information), the XPS survey spectrum of the γ‐In_2_Se_3_@rGO‐0.06 nanocomposite manifested the distinct characteristic signals of C, In, Se, and O elements. The elemental compositions were consistent with the results of XRD and Raman spectrum analyses discussed above. In the deconvoluted In 3d spectrum (Figure 3c), the peaks located at 444.6 eV and 452.0 eV were assigned to In 3d_2/5_ and In 3d_2/3_, respectively.^[^ ^48^ ^]^ The fine‐scanned Se 3d spectrum (Figure 3d) can be deconvoluted into two peaks at 54.1 eV and 55.6 eV, which were corresponded to Se 3d_5/2_ and Se 3d_3/2_ of Se^2−^.^[^ ^49^ ^]^ For the high‐resolution C 1s spectrum (Figure 3e), the peaks appearing at 285.2 eV, 286.4 eV, 288.6 eV, and 289.1 eV signified the existence forms of C = C/C─C, C─O, C = O, and O─C = O in the carbon components.^[^ ^50^, ^51^ ^]^

The pore structure characteristics of the γ‐In_2_Se_3_@rGO‐0.06 nanocomposite and bare In_2_Se_3_ were determined by N_2_ adsorption‐desorption isotherms. The bare In_2_Se_3_ presented a type III‐IV mixed isotherm with a small adsorption‐desorption hysteresis loop at high relative pressure, indicating the coexistence of macropores and mesopores, due to the stacking of the irregular In_2_Se_3_ particles which were interconnected by sintering.^[^ ^52^ ^]^ After the incorporation of the rGO matrix, a prominent pore structure change was observed. As is seen from Figure 3f that the γ‐In_2_Se_3_@rGO‐0.06 nanocomposite possessed a typical IV type isotherm. The emerged distinct H_3_ hysteresis loop in the high‐to‐medium pressure range (*P*/*P*_0_) of 0.45–0.95 was indicative of the existence of rich mesopores in materials. The corresponding pore size distribution curves further proved that the average size of mesopores in the bare In_2_Se_3_ shifts from ≈41.0 to ≈4.3 nm accompanying a higher pore distribution concentration after the proper addition of rGO. In addition, the Brunauer‐Emmett‐Teller (BET) specific surface area of the γ‐In_2_Se_3_@rGO‐0.06 nanocomposite and bare In_2_Se_3_ were determined to be 17.5 and 8.9 m^2^ g^−1^, respectively. These results all reflect the positive effect of the introduction of the rGO matrix, which can provide much improved dispersibility for In_2_Se_3_ NPs and properly enlarged specific surface for increased surface reaction sites and elevated Na^+^ diffusion ability.

### 2.2 Electrochemical Behaviors of the γ‐In_2_Se_3_@rGO Nanocomposites

**Figure 4:**
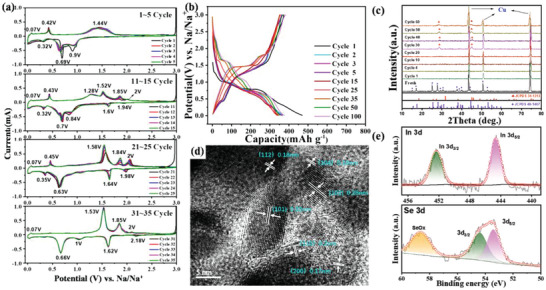
a) CV profiles of the γ‐In_2_Se_3_@rGO‐0.06 electrode. b) Galvanostatic discharge‐charge curves of the γ‐In_2_Se_3_@rGO‐0.06 electrode. c) The corresponding ex situ XRD patterns at different cycling times. d) HRTEM image and e) High‐resolution In 3d and Se 3d XPS spectra of the γ‐In_2_Se_3_@rGO‐0.06 electrode after 35 cycles at a current density of 0.1 A g^−1^.

The electrochemical behaviors of the various γ‐In_2_Se_3_@rGO nanocomposites and the bare In_2_Se_3_ were investigated in CR2025 coin‐type half cells with a sodium metal foil as the counter electrode. All the γ‐In_2_Se_3_@rGO electrodes displayed similar CV profiles (**Figure** **4**a; Figure S9a,b, Supporting Information) in the initial cathodic/anodic scans, signifying the analogous redox reaction processes. For the γ‐In_2_Se_3_@rGO‐0.06 electrode, two irreversible cathodic peaks, that emerged at 0.90 V and 0.69 V in the first scan, were attributed to electrolyte decomposition and the formation of a solid electrolyte interface (SEI) layer.^[^ ^53^ ^]^ From the second cycle on, three reversible peaks at 0.95 V, 0.64 V, and 0.32 V were clearly observed, which were the indications of the consecutive intercalation, conversion, and alloying reactions during the interaction between In_2_Se_3_ and Na^+^, respectively. Correspondingly, the sharp oxidation peak at 0.42 V and the broad one at 1.44 V in the anodic scans were ascribed to the stepwise desodiation reactions. The related reaction process is summarized below:^[^ ^36^ ^]^

(3) $${\mathrm{In}}_{2}{\mathrm{Se}}_{3}+\mathrm{xNa}+{\mathrm{xe}}^{-}\to {\mathrm{Na}}_{x}{\mathrm{In}}_{2}{\mathrm{Se}}_{3}\;$$

(4) $${\mathrm{Na}}_{x}{\mathrm{In}}_{2}{\mathrm{Se}}_{3}+\left(6-x\right){\mathrm{Na}}^{+}+\left(6-x\right)e^{-}\to 2\mathrm{In}+3{\mathrm{Na}}_{2}\mathrm{Se}\;$$

(5) $$\mathrm{In}+{\mathrm{xNa}}^{+}+{\mathrm{xe}}^{-}\to {\mathrm{Na}}_{x}\mathrm{In}\;$$

The ex‐situ XRD analysis at different discharge/charge states in the first cycle was conducted to verify the reaction process above. As displayed in Figure S10 (Supporting Information), the pristine XRD pattern of the γ‐In_2_Se_3_@rGO‐0.06 electrode showed obvious characteristic peaks of the γ‐In_2_Se_3_ phase. However, these sharp peaks totally disappeared when discharged to 1.5 V, which could be due to the Na‐insertion induced degradation of In_2_Se_3_ crystalline structure. With the further proceeding of discharging to 0.6 V, two weak peaks at 33.2 ^o^ and 39.4 ^o^ were distinguished. The diffraction signals could be assigned to (101) and (110) planes for tetragonal In species,^[^ ^54^ ^]^ suggesting the occurrence of conversion reaction (Equation 4). These peaks vanished subsequently and an inconspicuous peak at 33.5 ^o^, that could be indexed to Na_x_In, emerged when discharging to 0.01 V,^[^ ^36^ ^]^ corresponding to the alloying reaction (Equation 5). In the following charging process, the de‐alloying reaction was observed. Unfortunately, no peak signals were detected after charging to 3.0 V probably due to the poor crystallinity of the restored products. The ex situ XPS (Figure s11 , Supporting Information) measurements revealed the similar redox reaction behaviors to that discussed above. For the high‐resolution In 3d spectra, both the positions of In 3d_3/2_ and In 3d_5/2_ characteristic peaks gradually shifted to decreased bonding energies during the discharge process, and then returned back to the positions with higher bonding energies in the subsequent charge process, proving the reversible bonding state transition from In^3+^ to In^0^.

From Figure 4a, it should be noted that two new reduction peaks at 1.61 V and 1.94 V appeared during the repeated discharge/charge cycles (11th–15th cycle), and meanwhile the previous broad oxidation peak at 1.44 V was gradually split into four peaks at 1.28 V, 1.52 V, 1.85 V, and 2.01 V. The electrochemical reaction behaviors reflected in the CV curves of the present cycling stage were very close to that in the initial several CV curves for the bare In_2_Se_3_ electrode (Figure S9c, Supporting Information). The phenomenon could be related to the existence of the rGO matrix. By comparing the CV curves of the samples with different rGO contents (Figure 4a; Figure S9, Supporting Information), the redox reaction behaviors were more inclined to abide by the action mode of bare In_2_Se_3_ electrode with the decrease of rGO content. Since the lower rGO content means the increased agglomeration and sintering of adjacent In_2_Se_3_ NPs (Figure S4, Supporting Information), the pulverization phenomenon will be more serious. Thus, the evident difference of the initial electrochemical behaviors between the γ‐In_2_Se_3_@rGO‐0.06 electrode and the bare In_2_Se_3_ electrode could be resulted from the serious pulverization of the bare In_2_Se_3_ material (Figure S12a, Supporting Information). Nevertheless, the emergence of these new cathodic and anodic peaks in the CV curves of the γ‐In_2_Se_3_@rGO‐0.06 electrode since the 11th CV scan signified that the rGO matrix could only delay but not avoid the massive degradation of the In_2_Se_3_ NPs. As shown in the TEM image of the γ‐In_2_Se_3_@rGO‐0.06 nanocomposite after 10 discharge/charge cycles (Figure S12b, Supporting Information), obvious pulverization of In_2_Se_3_ NPs appeared. Fortunately, these crushed NPs still anchored on the surface of rGO, which maintained the structural integrity of electrode material.

The further pulverization of In_2_Se_3_ induced the continuous evolution of redox reaction behaviors. For example, in the CV curves from 21^st^ to 25^th^ scans, the cathodic peak at 1.64 V and the anodic peaks at 1.52 V, 1.85 V, and 2.01 V got stronger gradually, while the serial cathodic peaks at 0.70 V and 0.84 V were replaced by a more profound peak at 0.63 V. Meanwhile, the small anodic band at 1.28 V totally vanished away. The polytropic redox phenomenon with cycling suggests the complex transformation of oxidation/reduction behavior between In_2_Se_3_ and Na^+^ induced by the repeated sodiation/desodiation processes.

The electrochemical reaction evolution was terminated after around the 30th discharge/charge cycle. As can be seen in the CV curves belonging to 31st–35th cycles, only the cathodic peaks at 1.64 V and 0.66 V as well as the anodic peaks at 1.52 V and 1.85 V with high stability were conspicuous. The profiles were very different from the reaction feature shown in the initial CV cycles, indicating the intrinsic crystal structure change occurred. The galvanostatic discharge/charge curves for the γ‐In_2_Se_3_@rGO electrodes and the bare In_2_Se_3_ electrode (Figure 4b; Figure S13, Supporting Information) also demonstrated the similar sodiation/desodiation behavior, where the gradual transition of redox reaction plateaus during cycling was clearly observed while it maintained stably after 35 cycles. It is noted that the crystal structure evolution rate was dependent on current density. Figure S14 (Supporting Information) shows the discharge/charge curves of the γ‐In_2_Se_3_@rGO‐0.06 electrode at the current densities of 0.5 and 1.0 A g^−1^. The slower transition of redox reaction behavior with increasing current density is demonstrated, which could be resulted from the attenuate sodiation depth at higher current density and therefore the postponent particle pulverization.

### 2.3 Crystal Transformation Associated with the Sodiation/Desodiation Processes

The crystal phase transformation process of In_2_Se_3_ associated with the sodiation/desodiation was successfully traced by ex‐situ XRD observation. Figure 4c records the XRD patterns of the γ‐In_2_Se_3_@rGO‐0.06 electrode at different cycling times. It was revealed that the distinct peak signals belonging to γ‐In_2_Se_3_ for the fresh electrode totally disappeared after the 1st cycle, and maintained this In_2_Se_3_ signal‐free state with cycling until a perceptible tiny peak at 45.9 ^o^ appeared. The signal together with a steamed bun peak at 30.4 ^o^ became more significant in the diffraction pattern for the 30th cycle, in which the peaks at 30.4 ^o^ and 46.2 ° can be indexed to (102) and (104) planes of a β‐In_2_Se_3_ phase (JCPDS 34–1313, *a* = *b* = 4.01 Å, *c* = 9.64 Å) with a layered structure where indium atoms were placed in tetrahedral and octahedral interstices (Figure S15, Supporting Information).^[^ ^55^ ^]^ The as‐formed β‐In_2_Se_3_ phase was further verified by HRTEM observation and XPS measurement. As can be seen in Figure 4d, the HRTEM image of the γ‐In_2_Se_3_@rGO‐0.06 nanocomposite after suffering 35 discharge/charge cycles revealed the lattice fringes with the determined layer distances of 0.20, 0.28, and 0.32 nm, which were corresponded to the (110), (102), and (101) planes of β‐In_2_Se_3_ (JCPDS 34–1313), respectively. Synchronously, the XPS survey (Figure S16, Supporting Information) and the high‐resolution In 3d and Se 3d spectra (Figure 4e) of the cycled electrode were analogous to that of the γ‐In_2_Se_3_@rGO‐0.06 nanocomposite before the electrochemical tests. It is noteworthy that the peak positions belonging to the deconvoluted In 3d_5/2_, In 3d_3/2_, Se 3d_5/2_, and Se 3d_3/2_ upshifted to the increased binding energy, which could be resulted from the changes of coordination environment of atoms and crystal cell parameter variation after the crystal transformation from γ to β In_2_Se_3_ phase.^[^ ^36^, ^56^ ^]^

According to the previous studies, the β phase of In_2_Se_3_ was generally considered to be hard to stably exist at room temperature in bulk materials, and its stable crystalline phase characteristic was only observed when heating the α phase to 200 °C or rising the pressure to 0.7 GPa.^[^ ^57^, ^58^ ^]^ However, when the crystal size of In_2_Se_3_ is diminished to nanoscale, such as ultrasmall nanoparticles and ultrathin nanofilms, the mingled β phase with α phase or pure β phase were detected and persisted at room temperature. Gu et al.^[^ ^59^ ^]^ reported a 4–5 nm thick single‐crystal In_2_Se_3_ film which was obtained by means of mechanical exfoliation onto Si_3_N_4_/Si or SiO_2_/Si substrates. It was concluded that the stabilization of β phase during the phase transformation process from α phase might be attributed to the nano‐size effect, which resulted in the formation of superstructure that represented a periodic distortion in lattices and frustrated the reversible phase transition back to α phase. Therefore, in our present study, even though the phase transformation behavior from γ to β phase is unusual, it seems reasonable since the repeated sodiation/desodiation led to the continuous pulverization of In_2_Se_3_ nanoparticles to the dimension of less than 10 nm that triggered the irreversible phase transformation during the electrochemical redox reaction process.

### 2.4 Effect of Crystal Transformation on Sodium Storage Performance of the γ‐In_2_Se_3_@rGO Electrodes

**Figure 5:**
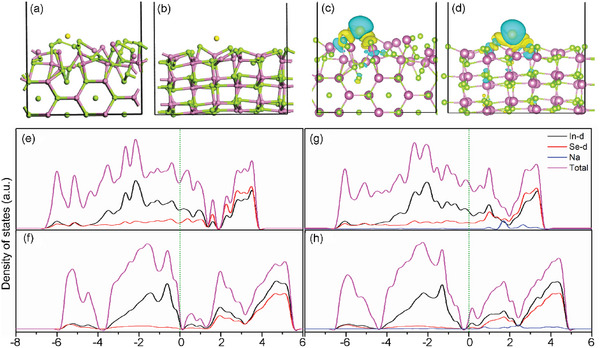
The optimized structure of a) Na/β‐In_2_Se_3_ and b) Na/γ‐In_2_Se_3_. c,d) The isosurface of charge density difference between Na/β‐In_2_Se_3_ and Na/γ‐In_2_Se_3_. The blue isosurfaces are regions of negative charge accumulated and the yellow isosurfaces are regions of positive charge accumulated. The purple, green, and yellow spheres denote In, Se, and Na atoms, respectively. The DOS of β‐In_2_Se_3_ e), γ‐In_2_Se_3_ f), Na/β‐In_2_Se_3_ g), and Na/γ‐In_2_Se_3_ h), respectively.

To investigate the effect of the special phase transformation on the electronic conductivity of crystalline structures and their association behaviors with sodium, which are closely related to the electrode performance, first‐principle calculations are carried out. The adsorption energies (E_ad_) of Na atom on the (110) plane of β and γ‐In_2_Se_3_ were calculated. As shown in **Figure** **5**a,b, the binding energies of Na/β‐In_2_Se_3_ and Na/γ‐In_2_Se_3_ are −5.66 eV and −2.16 eV, respectively, which indicates that the binding of Na atom on β‐In_2_Se_3_ was more accessible and had better stability for the sodiation structure. Furthermore, the electronic conductivity of the clean substrate for β and γ‐In_2_Se_3_ (110) and the corresponding Na‐doped products was evaluated by charge density difference and density of states (DOS) analyses. The charge density differences of β‐Na and γ‐Na are shown in Figure 5c,d. The negative charge accumulates on the Na atom in Na/β‐In_2_Se_3_ and Na/γ‐In_2_Se_3_, and the positive charge accumulates around the Se and In atoms in Na/β‐In_2_Se_3_, while only around Se atom in Na/γ‐In_2_Se_3_. The difference of charge transfer between Na/β‐In_2_Se_3_ and Na/γ‐In_2_Se_3_ indicates that both of them are electrically conductive but the charge transfer in Na/β‐In_2_Se_3_ is stronger. The density of states (DOS) for β‐In_2_Se_3_ (e), γ‐In_2_Se_3_ (f), Na/β‐In_2_Se_3_ (g), and Na/γ‐In_2_Se_3_ (h) are shown in Figure 5e–h. All of them are conductors with the valence bands crossing through the Fermi level. The d‐orbitals in Se atoms mainly contribute to the conductivity. However, their differences deserve special attention. The comparison between 5e and 5g reflects that the adsorption of Na atom enhances the electronic density, especially around the Fermi level, while the variation of electronic density for γ‐In_2_Se_3_ is not obvious (5f and 5h). The difference between 5g and 5h show that the electronic states and density of the former are more stronger than the latter, which further prove the better conductivity of Na/β‐In_2_Se_3_. Besides, compared to Na/γ‐In_2_Se_3_, the integration of Na enhances the conductance of β‐In_2_Se_3_, as more electronic states appeared in conduction band (1.3–3.5 eV).

**Figure 6:**
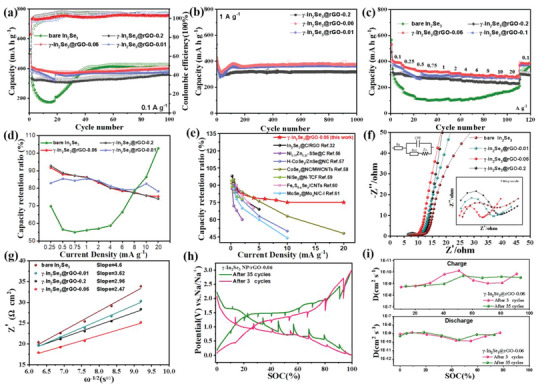
Electrochemical performance of the γ‐In_2_Se_3_@rGO electrodes and the bare In_2_Se_3_ electrode: a) Cycling performance, b) Long‐term cycling ability, c) Rate capability. d) The relationship between rate capacity retention and current density. e) Performance comparison of the γ‐In_2_Se_3_@rGO‐0.06 electrode with the recently reported MS materials used in SIBs. f) Nyquist plots with the fitted lines by using the inset equivalent circuit, g) Real parts of the complex impedances versus ω^−1/2^ (angular frequency). h,i) GITT curves and the values of $D_{{\mathrm{Na}}^{+}}$D at different discharge/charge states for the γ‐In_2_Se_3_@rGO‐0.06 electrode after 3 and 35 cycles.

The improved electrical conductivity and association conditions of In_2_Se_3_ with sodium after the γ → β phase transformation contributed to prominently enhanced electrochemical performance. **Figure** **6**a shows the cycling performance of γ‐In_2_Se_3_@rGO electrodes and the bare In_2_Se_3_ electrode at a current density of 0.1 A g^−1^. The discharge/charge capacities of the bare In_2_Se_3_, γ‐In_2_Se_3_@rGO‐0.01, γ‐In_2_Se_3_@rGO‐0.06 and γ‐In_2_Se_3_@rGO‐0.2 electrodes were 534/341, 526/387, 511/426, and 499/347 mA h g^−1^ in the first cycle with initial Coulombic efficiencies (ICEs) of 63.9%, 73.4%, 83.3%, and 69.5%, respectively. The higher initial reversible capacity and ICE of the γ‐In_2_Se_3_@rGO‐0.06 electrode were resulted from the high loading amount of In_2_Se_3_ NPs and their even dispersion on rGO matrix.^[^ ^60^ ^]^ For the electrochemical behavior in bare In_2_Se_3_ electrode, it is noted that rapid capacity decay within the initial 20 cycles occurred due to the serious pulverization of In_2_Se_3_ NPs as demonstrated by TEM observation discussed above. Interestingly, the reversible capacity gradually elevated with the further cycling. After 60 cycles, a high discharge capacity of 427 mA h g^−1^ was received and retained stably in the following cycles. In view of the distinct evolution of redox reaction behavior during discharge/charge process, it is believed that the recapture of high reversible capacity and the substantially elevated cycling stability could be attributed to the featured γ → β phase transformation of In_2_Se_3_, for which the high conductivity of γ‐In_2_Se_3_ and its better accessibility to Na^+^ facilitated the charge transfer and electrochemical reaction kinetics in electrode materials. After the combination with rGO matrix, the initial capacity decay phenomenon was greatly suppressed with the increased proportion of rGO in the composites, manifesting the rGO matrix effectively buffered the volume fluctuation of γ‐In_2_Se_3_ and kept electrode structure from being disintegrated during sodiation/desodiation processes. In addition, due to the progressive phase transition process of In_2_Se_3_ accompanying particle pulverization, CE of the electrodes presented distinct fluctuation with cycling (Figure S17, Supporting Information). And the higher content of rGO contributed to better performance and stability of CE.

Among the three rGO‐involved composite electrodes, the γ‐In_2_Se_3_@rGO‐0.06 electrode exhibited both the highest specific capacity and the best cycling stability, and an attractive discharge capacity of 411 mA h g^−1^ (corresponding to the energy density of 443 Wh kg^−1^) was maintained after 100 cycles. Moreover, in the test condition at a large current density of 1.0 A g^−1^, the γ‐In_2_Se_3_@rGO electrodes all exhibited the similar cycling behaviors, in which the behavior that a sudden capacity drop and then restored to an ultrastable value in the initial cycling stage was observed. The behavior could be originated from the slow γ → β phase transformation dynamics of In_2_Se_3_. Anyhow, these hybrid electrodes displayed superior long‐term cycling ability. Typically, as shown in Figure 6b, even after suffering from 1000 cycles at 1.0 A g^−1^, a high reversible capacity of 378 mA h g^−1^ was still retained for the γ‐In_2_Se_3_@rGO‐0.06 electrode with a low capacity loss ratio of 0.011% per cycle. Furthermore, the long‐term cycling performances at 2.0 A g^−1^ and 4.0 A g^−1^ (Figure S18, Supporting Information) were also parallel to that at 1.0 A g^−1^, verifying the superior electrochemical stability and the robustness of the β‐In_2_Se_3_ dominated electrode structure. The high reliability of β‐In_2_Se_3_@rGO configuration was further verified by SEM observation of the tested electrode. As is seen in Figure S19 (Supporting Information), a rough surface that is uniformly covered by a thick SEI layer can be distinguished for the γ‐In_2_Se_3_@rGO‐0.06 electrode after suffering 1000 cycles. The rugged morphology was probably formed during the pulverization stage of the phase evolution process. It is fortunately revealed that no obvious microcracks were discovered, and the uniform dispersion of active materials ensured the satisfactory electrochemical cycling stability.

The rate capabilities of the electrode materials were also investigated to explore the application potential in high‐power devices. It is noted from Figure 6c that the bare In_2_Se_3_ electrode presented a “roller coaster”‐like rate behavior with the stepwise increased current densities due to the existence of γ → β phase transformation. However, compared to the electrochemical behavior shown in the initial cycling process at 0.1 A g^−1^, an obvious prolongment of cycling times for the capacity drop was observed, further proving the sluggish phase transformation dynamics that probably dominated by the particle size of In_2_Se_3_ NPs. But what threshold of nanoparticle size or pulverization degree of In_2_Se_3_ will trigger the phase transformation needs to be carefully determined in the subsequent works.

For the γ‐In_2_Se_3_@rGO electrodes, the very positive effect of the rGO matrix on stabilizing the integrated electrode structure was further verified. The γ‐In_2_Se_3_@rGO‐0.01 electrode presented analogous initial electrochemical behavior to that in the bare In_2_Se_3_ electrode due to the insufficient proportion of rGO. With the further increased content of rGO, the hybrid electrode materials exhibited steadily enhanced rate performance. Remarkably, with the current densities increased from 0.1 to 0.25, 0.5, 0.75, 1.0, 2.0, 4.0, 6.0, 8.0, and 10.0 A g^−1^, the γ‐In_2_Se_3_@rGO‐0.06 electrode exhibited the reversible capacities of 376, 340, 327, 324, 320, 309, 297, 291, 284, and 280 mA h g^−1^. Even at the ultrahigh current density of 20.0 A g^−1^, a high reversible capacity of 272 mA h g^−1^ was still achieved. Moreover, the reversible capacity was immediately restored to 395 mA h g^−1^ when the current density was reset back to 0.1 A g^−1^. The results manifest the excellent rate ability, which could be ascribed to the synergistic effect between the rGO matrix and the β phase structure for constructing an ultrastable hybrid configuration and promoting fast electrochemical reaction response upon increased current densities. In addition, the rate capacity retention of the γ‐In_2_Se_3_@rGO electrodes shown in Figure 6d more intuitively reflects the prominent role of the rGO matrix for the substantially enhanced rate performance even at the low rGO content of 3.15 wt.% (corresponding to the γ‐In_2_Se_3_@rGO‐0.01 electrode), and the added content of rGO surpassed 15.78 wt.% seems no further effect for the performance enhancement any more. The rate performance of the γ‐In_2_Se_3_@rGO‐0.06 electrode was also compared with the typical MS electrodes in SIBs reported recently (Figure 6e; Table S3, Supporting Information).^[^ ^37^, ^61^, ^62^, ^63^, ^64^, ^65^, ^66^ ^]^ The distinct advantage in rate capacity retention especially at high current densities signifies the great application potential of β‐In_2_Se_3_ based electrode materials for high‐power SIBs.

EIS, GITT as well as the CV curves at different scan rates were conducted to investigate the kinetics behaviors of the γ‐In_2_Se_3_@rGO electrodes. Figure 6f displays the Nyquist plots of the γ‐In_2_Se_3_@rGO electrodes with different rGO content. Generally, the semicircle in the high‐medium frequency region and the inclined line in the low frequency region are corresponding to charge transfer resistance (*R*_ct_) and Warburg impedance (*Z*_w_), respectively.^[^ ^62^ ^]^ Z_w_ is related to Na^+^ diffusion efficiency, which is in direct proportion to Warburg factor (*σ*) according to the relationship: Z′ = R_D_  + R_L_ + σω^−1/2^(Figure 6g).^[^ ^67^ ^]^ The plots were fitted based on the equivalent circuit shown in the inset of Figure 6f, and the obtained values of *R*_ct_ and *σ* for the electrode materials were listed in Table S4 (Supporting Information). The results manifest that the γ‐In_2_Se_3_@rGO‐0.06 electrode had both the lowest values of *R*_ct_ (1.45 Ω) and the smallest *σ* (2.47) among the three composite electrodes and the bare In_2_Se_3_ electrode, suggesting its more reasonable structural hybridization between In_2_Se_3_ and rGO with optimized rGO content. EIS of the γ‐In_2_Se_3_@rGO‐0.06 electrode after 35 cycles was also measured, and the Nyquist plots (Figure S20, Supporting Information) presented two semicircles in high‐ and medium‐frequency region, which are related to SEI resistance (*R*_sf_) and *R*_ct_.^[^ ^68^ ^]^ After fitted by a proper equivalent circuit (Inset of Figure S20, Supporting Information), the resistance value of R_sf_+R_ct_ can maintain a low level (4.68 Ω), inferring the favorable conductivity of the γ‐In_2_Se_3_@rGO‐0.06 electrode after suffering serious particle pulverization, probably benefited from the crystal transformation of In_2_Se_3_. The diffusion coefficients ($D_{{\mathrm{Na}}^{+}}$D) in the γ‐In_2_Se_3_@rGO electrodes were further estimated to evaluate the Na^+^ transportation behaviors.^[^ ^69^ ^]^ The calculated $D_{{\mathrm{Na}}^{+}}$ of the γ‐In_2_Se_3_@rGO‐0.06 electrodes before and after 35 cycles are roughly equivalent (1.89 × 10^−13^ and 1.19 × 10^−13^ cm^2^ s^−1^ respectively), which indicates that the pulverization of active matter and the repeatedly formed SEI layer did not result in the deterioration of Na^+^ diffusion kinetics. The Na^+^ diffusion ability in the γ‐In_2_Se_3_@rGO‐0.06 electrodes before and after 35 cycles was further studied by GITT analysis basedon the following equation,^[^ ^70^ ^]^ in which L, τ, ΔE_s_ and ΔE_τ_ are Na^+^ diffusion distance, constant current titration time, current pulse induced voltage change and the total voltage change excluding *iR* drop, respectively.

(6) $$D_{{\mathrm{Na}}^{+}}=\frac{4L^{2}}{\pi \tau }\;{\left(\frac{{\Delta E}_{s}}{{\Delta E}_{\tau }}\right)}^{2}\;$$

From the tested results and the calculated $D_{{\mathrm{Na}}^{+}}$ data (Figure 6h,i), it is revealed that the Na^+^ diffusion rate for the hybrid electrodes before and after 35 cycles had similar variation trends during the sodiation/desodiation process, and the $D_{{\mathrm{Na}}^{+}}$ values at different discharge/charge states were basically in the same level for the two electrodes. The slightly decreased and increased $D_{{\mathrm{Na}}^{+}}$ at ≈1.5 V and 2.4 V respectively during charge process for the cycled electrode could be due to the different redox reaction mechanism. The CV curves at different scan rates were gathered and analyzed to further study the sodium storage behaviors of the In_2_Se_3_/rGO‐0.06 electrodes before and after the crystal transformation. As shown in Figure S21a,b (Supporting Information), with the augment of scan rates, the positions of the redox reaction peaks kept still well for both of the electrodes, indicating the low polarization and quick dynamics response. The power‐law relationship: *i*  =  *av^b^* is usually employed to assess the electrochemical reaction kinetics characteristics of active materials,^[^ ^71^ ^]^ where *i* and *v* are peak currents and scan rate, respectively. Generally, the *b* values are in the range between 0.5 and 1.0, where the value close to 1.0 denotes a capacitive reaction process, while the value approaches to 0.5 means a diffusion‐dominated sodium storage behavior. As revealed in Figure S21c,d (Supporting Information), the *b* values of the redox peaks for the two electrodes far exceed 0.5 in most of cases, indicating the sodium storage mechanism is mainly originated from capacitive behaviors, which accounts for the fast electrochemical reaction kinetics and excellent rate capability.

**Figure 7:**
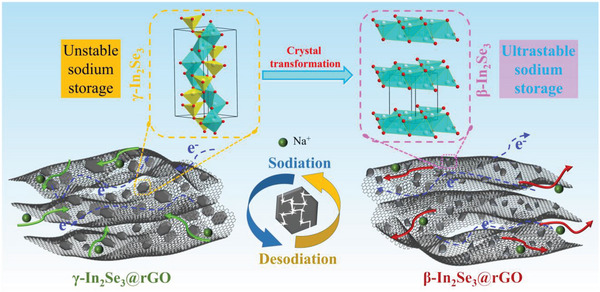
Schematic illustration of the structural evolution in the γ‐In_2_Se_3_@rGO electrodes during sodiation/desodiation processes for substantially enhanced electrochemical performance.

Based on the results of material characterizations and electrochemical behavior analyses above, the greatly improved sodium storage performance of the γ‐In_2_Se_3_@rGO‐0.06 electrode is attributed to the favorable synergistic effect between the introduction of rGO as a matrix and the γ → β phase transformation of In_2_Se_3_. As schematically depicted in **Figure** **7**, even though the repeated sodiation/desodiation resulted in the serious pulverization of In_2_Se_3_, the existence of rGO matrix effectively buffered the volume fluctuation and provided adhesion sites for the fractured active nanoparticles. Thus stable electrode structure integrality was achieved, contributing to the high reversible capacity and superior cycling performance. More importantly, the continuous dimension decrease of the In_2_Se_3_ particles during the redox reaction processes triggered the irreversible phase transformation of In_2_Se_3_ from γ to β phase. The high conductivity of β‐In_2_Se_3_ and its better accessibility to Na^+^ further facilitated the accelerated charge transfer and ion diffusion kinetics, which conduced to the substantially enhanced long‐term cycling stability and excellent rate capability.

Finally, to explore the practical application potential of the γ‐In_2_Se_3_@rGO electrodes, Na_3_V_2_(PO_4_)_3_ cathodes were coupled to assemble full cells and the electrochemical performances were evaluated. The as‐prepared Na_3_V_2_(PO_4_)_3_ cathode in half‐cell released a high discharge capacity of 106 mA h g^−1^, and exhibited a favorable cycling stability (Figure S22, Supporting Information). Correspondingly, the γ‐In_2_Se_3_@rGO‐0.06//Na_3_V_2_(PO_4_)_3_ full cell also manifested a satisfactory electrochemical performance. Based on the mass of anode material, the initial charge/discharge capacities are 368/315 mA h g^−1^ with an initial Coulombic efficiency of 85.6% (Figure S23a, Supporting Information), and the calculated energy density considering the total mass of electrode materials is 379 W h kg^−1^. The cycling and rate performances are also attractive (Figure S23b–d, Supporting Information). Remarkably, with the stepwise rise of current densities from 0.1 to 0.25, 0.5, 0.75, 1.0, and 2.0 A g^−1^, the discharge capacities of 316, 305, 290, 282, 271, and 266 mA h g^−1^ are achieved, suggesting the high rate capability and promising application potential of this battery system for high‐power SIBs.

## 3 Conclusion

In summary, a unique irreversible crystal transformation of In_2_Se_3_ from γ to β phase was discovered during the repeated discharge/charge processes in SIBs, which was demonstrated to be resulted from the sodiation/desodiation induced persistent dimension degradation of In_2_Se_3_ particles. γ‐In_2_Se_3_/reduced graphene oxide (γ‐In_2_Se_3_@rGO) composites as a prototype were prepared through a facile wet chemistry method to systematically investigate the phase transition behavior, the effect of the introduction of rGO matrices and their couple effect on electrochemical performance. The material characterization and electrochemical evaluation results indicated the profound influence of the two factors. Especially, the content of rGO as a matrix played important roles in improving the dispersibility of In_2_Se_3_ and suppressing the pulverization phenomenon during redox reaction process. Even though the serious pulverization of In_2_Se_3_ after suffering the repeated sodiation/desodiation occurred, the active materials still anchored on the surface of rGO to maintain the integrity of electrode structure. The continuous particle pulverization of In_2_Se_3_ accompanied the gradual γ → β phase transition. The first‐principle calculations revealed the highly beneficial effect of the γ → β phase transformation, which results in the improved conductivity of In_2_Se_3_ and the more facilitated association inclination with sodium. Benefited from the prominent synergistic effect between the proper introduction of rGO and the crystal transformation, the γ‐In_2_Se_3_@rGO‐0.06 electrode exhibited substantially elevated sodium storage performance, especially the ultra‐stable cycling ability and excellent rate performance in both half and full batteries. The kinetics analysis results demonstrate the distinctly accelerated charge transfer and ion diffusion efficiency, which are responsible for the improved electrochemical performance.

## 4 Experimental Section

### Materials

All of the chemicals were analytical grade and directly used without further purification. GO was prepared by means of a modified pressurized oxidation method with natural graphite (purity>99%, 200 mesh) as the raw material.

### Preparation of the In(OH)3 NPs

The In(OH)_3_ NPs were prepared via a solvothermal route. First, 0.6 g Polyvinylpyrrolidone (PVP) was added into 38 mL of the mixed solvent of N,N‐dimethylformamide (DMF) and distilled water (1:1 in volume ratio), and ultrasonically dispersed to form a uniform solution. After that, 0.2 g In(NO_3_)_3_·4H_2_O was further added in and magnetically stirred for 0.5 h. Then the mixed solution was transferred into a PTFE‐lined autoclave (50 mL) and reacted at 120 °C for 10 h. The as‐obtained gray sediment was collected by centrifugation and rinsed with distilled water for three times. After vacuum freeze drying, the In(OH)_3_ NPs were obtained.

### Preparation of the γ‐In2Se3@rGO Nanocomposites

In(OH)_3_ NPs(0.1 g) and x g GO (*x* = 0.2, 0.06, and 0.01) were added into 100 mL distilled water, and ultrasonically treated for 1 h to form a homogeneous suspension. Subsequently, the suspension was quickly frozen by suffering from a liquid nitrogen quenching. The frozen solid was then freeze‐dried to obtain the In(OH)_3_@GO composites. After that, the composite and selenium powder was mixed at a weight ratio of 1:6 and heat‐treated at 600 °C for 3 h in an Ar‐protected tube furnace to obtain the resulting γ‐In_2_Se_3_@rGO nanocomposites. The as‐prepared composites with the GO's feeding amounts of 0.2 g, 0.06 g, and 0.01 g were denoted as γ‐In_2_Se_3_@rGO‐0.2, γ‐In_2_Se_3_@rGO‐0.06, and γ‐In_2_Se_3_@rGO‐0.01, respectively. For a comparison, the bare In_2_Se_3_ sample was prepared by the same selenization method without the involvement of GO.

### Material Characterizations

The phase composition and crystallinity of the prepared samples were determined by powder X‐ray diffraction diffractometer (XRD, Bruker D8 Advance) and a Raman spectroscopy (Renishaw RM2000 confocal) with an excitation line of 514 nm. The morphology and texture of the samples were observed by scanning electron microscopy (SEM, JIOL, JSM‐7001F) and high‐resolution transmission electron microscopy (HRTEM, JIOL, JIM 2010) with an energy dispersive X‐ray (EDX) spectroscopy. The surface elemental composition and bonding state were explored by X‐ray photoelectron spectroscopy (XPS, Thermo ESCALAB 250). The BET (Brunauer‐Emmet‐Teller) specific surface area and pore information were characterized by N_2_ adsorption/desorption tests at 77 K (Micrometritics ASAP 2020). The content of In_2_Se_3_ in the samples was estimated by thermogravimetric (TG) analysis (NETZSCH, STA449F3) in air atmosphere. The electric conductivity of the samples was measured through four‐probe method on a powder resistance meter (ROOKO, FT‐100E).

### Electrochemical Measurements

The electrochemical tests were evaluated in CR2025 coin‐type half cells with a sodium foil disc as the reference/counter electrode. The working electrodes were fabricated by mixing the as‐prepared samples (80 wt.%), super P (10 wt.%) as the conductive agent, and polyvinylidene fluoride (10 wt.%) as the binder in drops of N‐methylpyrrolidone to form a uniform slurry, and then coated on a copper foil which subsequently baked in a vacuum oven at 120 °C overnight. The electrode discs with a diameter of 12 mm and area mass loading of ≈1.3 mg cm^−2^ were obtained by punching. The electrolyte and separator were 1 m NaPF_6_ in Diglyme and glass fiber membrane (Whatman), respectively. The galvanostatic discharge/charge tests and galvanostatic intermittent titration (GITT) measurement were performed on a CT2001A (LAND, Wuhan) battery test system in the voltage range from 0.01 to 3.0 V. The cyclic voltammetry (CV) was measured by an electrochemical workstation (CHI660E) in the potential window of 0.01–3.0 V at a scan rate of 0.25 mV s^−1^. The electrochemical impedance spectroscopy (EIS) analyses were carried out over a frequency range of 100 kHZ‐0.01 HZ. For the assembly of full cells, a Na_3_V_2_(PO_4_)_3_ cathode material was synthesized by using a modified citric acid sol‐gel method,^[^ ^72^ ^]^ and the corresponding electrode was fabricated by casting a slurry containing 80 wt.% Na_3_V_2_(PO_4_)_3_, 10 wt.% super P, and 10 wt.% PVDF on an Al foil. The mass loading ratio between the cathode and anode was approximately 3:1.

### Computational Methods

The first‐principle calculations were carried out using the Vienna ab initio simulation package (VASP).^[^ ^73^ ^]^ The projector augmented wave (PAW)^[^ ^74^, ^75^ ^]^ pseudopotential was employed to obtain the cohesive energies of Na. The Perdew–Burke–Ernzerhof (PBE) function was used for describing exchange and correlation.^[^ ^76^ ^]^ The cutoff energy was set as 400 eV, which gives well‐converged relative energies for the system. The structural optimizations for β and γ phase of In_2_Se_3_ and the corresponding doped products with Na atom were done when the force tolerance and the energy difference were lower than 0.02 eV Å^−1^ and 10^−5^ eV, respectively. The vacuum layer was set over 20 Å to avoid the slab‐to‐slab interaction. And the bottom layer was fixed to its bulk geometry during optimization and other atoms were fully relaxed. The 6 × 6 × 1 k‐point meshes were chosen for geometry optimizations and the following calculations on electronic properties. The adsorption energy (*E*_ad_) of Na atom was evaluated according to E_ad_ = E_total_‐E_slab_‐E_Na_, in which the E_slab_, E_Na_, and E_total_ represented the energy of clean substrate, Na atom and the adsorption system (Na/β‐In_2_Se_3_ or Na/γ‐In_2_Se_3_), respectively.

## Conflict of Interest

The authors declare no conflict of interest.

## Supporting information

Supporting Information

## Data Availability

Research data are not shared.
