# Supplementary material for: Synergistic γ‐In2Se3@rGO Nanocomposites with Beneficial Crystal Transformation Behavior for High‐Performance Sodium‐Ion Batteries
Source: Adv Sci (Weinh). 2023 Aug 4;10(28):2303108. doi: 10.1002/advs.202303108 (PMC10558666; doi:10.1002/advs.202303108)
Supplement: Supplementary file 1 — Supporting Information [file ADVS-10-2303108-s001.pdf]

ADVANCED SCIENCE
Open Access

Supporting Information

for *Adv. Sci.*, DOI 10.1002/advs.202303108

Synergistic γ-In_2Se_3@rGO Nanocomposites with Beneficial Crystal Transformation Behavior for High-Performance Sodium-Ion Batteries

Yun Zhao, Haoyue Zhang, Yong Li, Canliang Ma, Wenjuan Tian*, Xingguo Qi, Gaoyi Han and Zongping Shao**

Supporting Information

Synergistic γ-In_2Se_3@rGO nanocomposites with beneficial crystal transformation behavior for high-performance sodium-ion batteries

Yun Zhao, Haoyue Zhang, Yong Li, Canliang Ma, Wenjuan Tian*, Xingguo Qi, Gaoyi Han, Zongping Shao**

Y. Zhao, H. Y. Zhang, C. L. Ma, W. J. Tian, G. Y. Han
Institute of Molecular Science, Key Laboratory of Materials for Energy Conversion and Storage of Shanxi Province, Key Laboratory of Chemical Biology and Molecular Engineering of Education Ministry
Shanxi University
Taiyuan 030006, P.R. China
Y. Zhao
Shanxi-Zheda Institute of Advanced Materials and Chemical Engineering
Taiyuan 030006, P.R. China
Y. Li
Research Center for Fine Chemicals Engineering
Shanxi University
Taiyuan 030006, P.R. China
X. G. Qi
Shanxi Huana Carbon Energy Technology Co., Ltd.
Taiyuan 030006, P.R. China
Z. P. Shao
WA School of Mines: Minerals Energy and Chemical Engineering (WASM-MECE)
Curtin University
Perth, WA 6102, Australia
E-mail: zongping.shao@curtin.edu.au; zhaoyun@sxu.edu.cn; tianwenjuan@sxu.edu.cn.

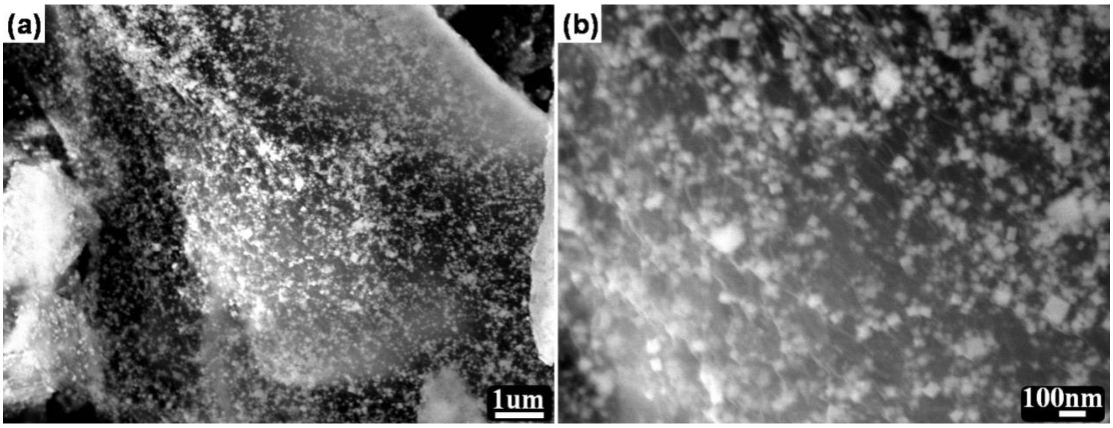

Figure S1 (a) SEM images of the $In(OH)_3$@GO composite.

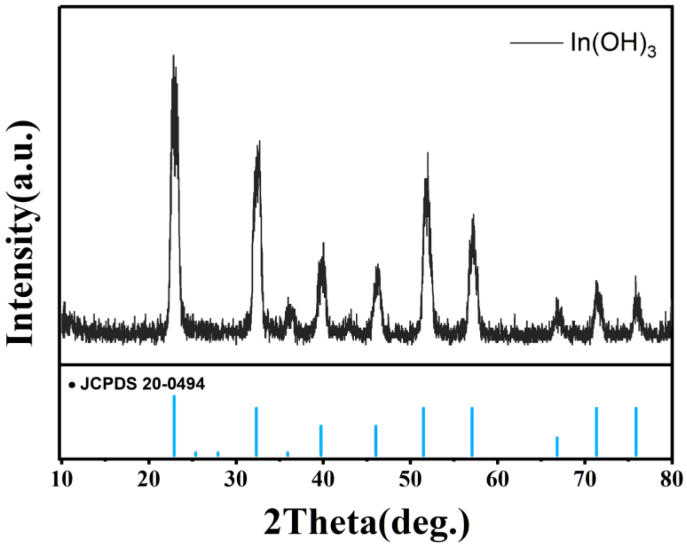

Figure S2 XRD pattern of the $In(OH)_3$@GO composite.

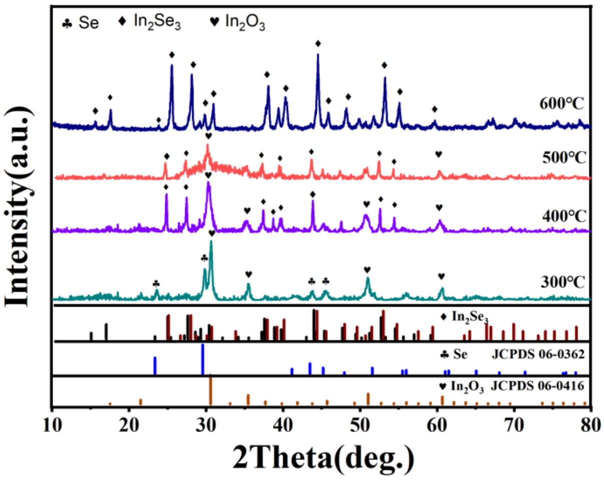

Figure S3 XRD patterns of the products at different selenization temperature.

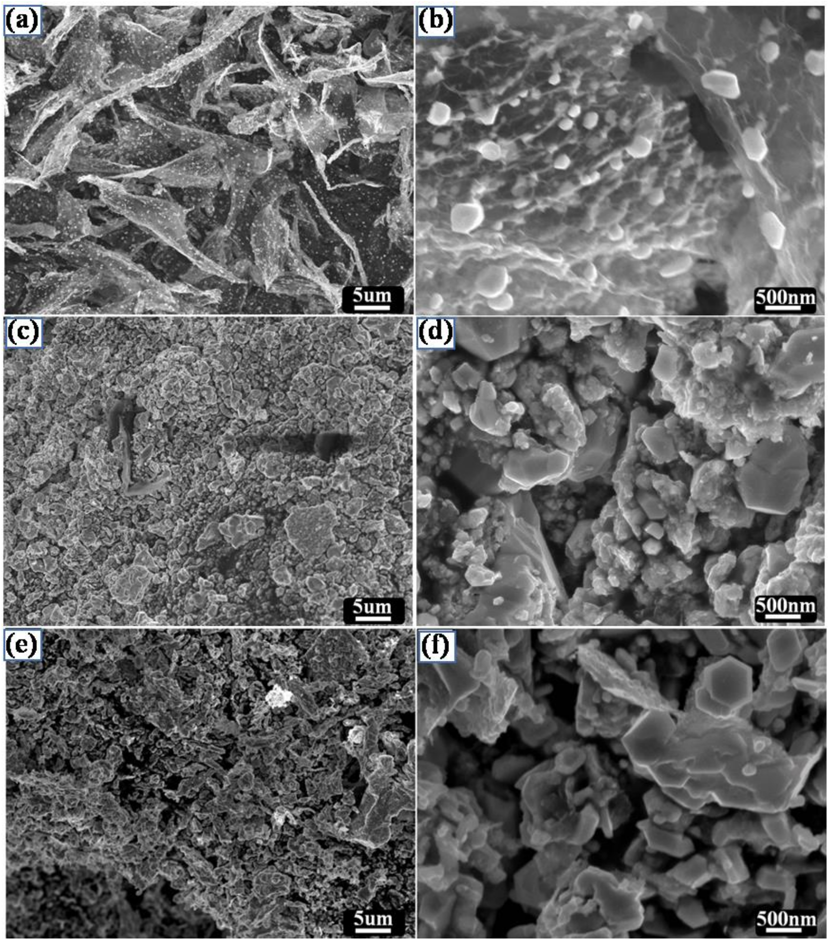

Figure S4 (a)-(b) SEM images of the γ-In_2Se_3@rGO-0.2, (c)-(d) γ-In_2Se_3@rGO-0.01 and (e)-(f) the bare In_2Se_3, respectively.

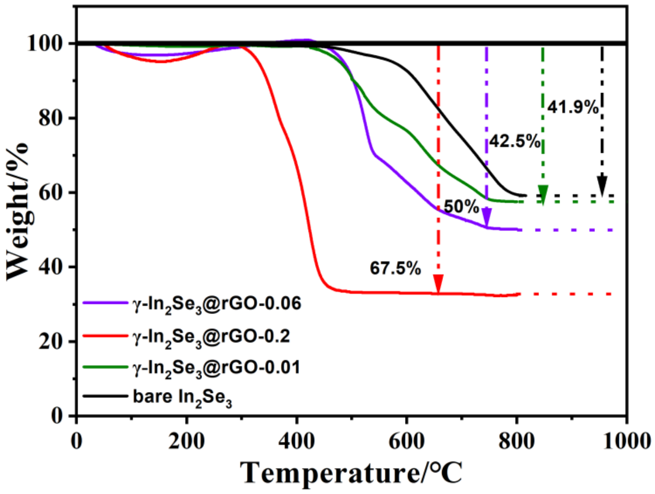

Figure S5 TG curves of the γ-In_2Se_3@rGO nanocomposites with different In_2Se_3 weight contents.

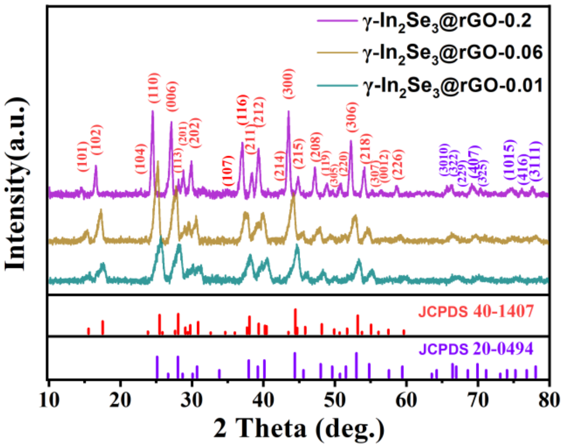

Figure S6 XRD patterns of the γ-In_2Se_3@rGO nanocomposites with differerent rGO content.

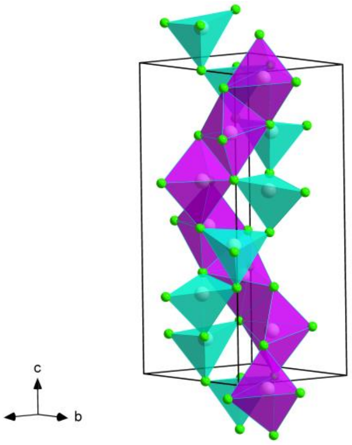

Figure S7 The helical structure in crystal cell of γ-In_2Se_3 (JCPDS 40-1407, a=b=7.13 Å, c=19.38 Å): (a) Side view; (b) Top view.

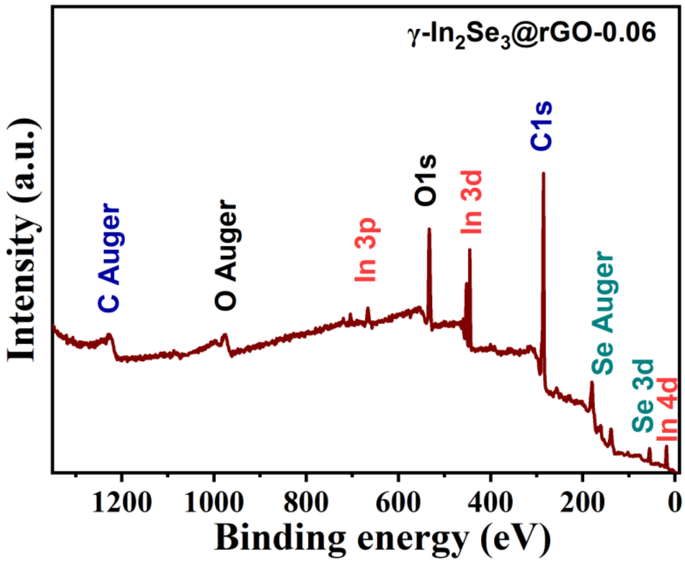

Figure S8 XPS survey spectrum of the γ-In_2Se_3@rGO-0.06 nanocomposite.

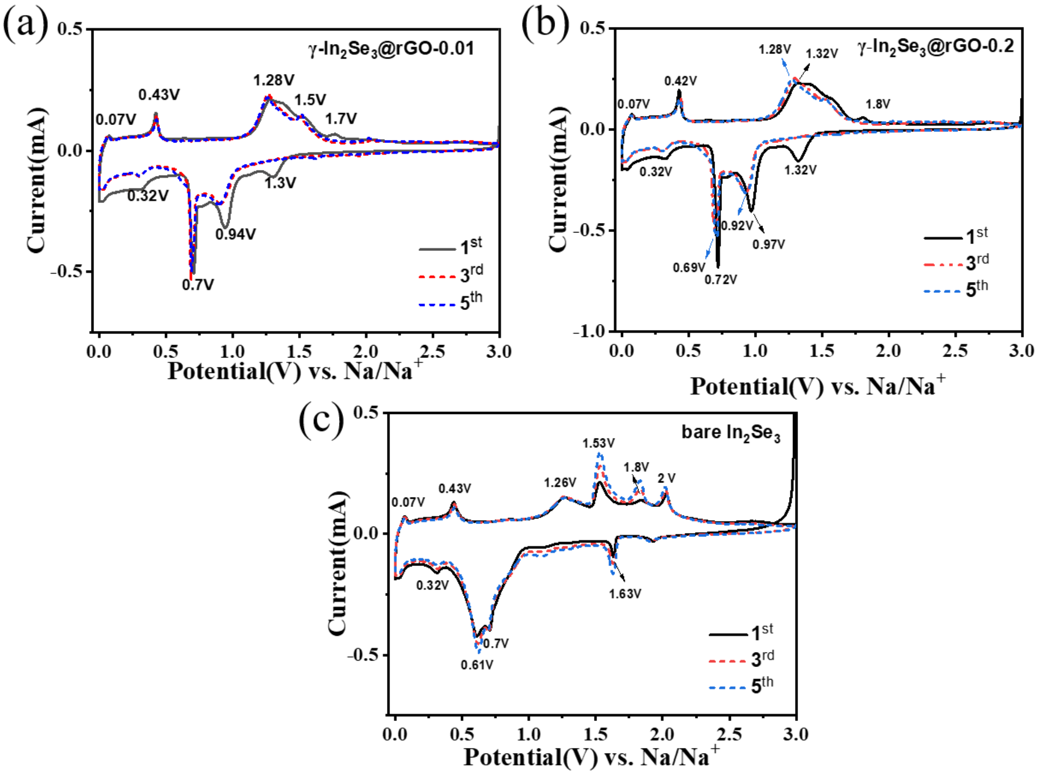

Figure S9 CV and galvanostatic discharge-charge curves of the γ-In_2Se_3@rGO-0.2, γ-In_2Se_3@rGO-0.01 and the bare In_2Se_3 electrodes.

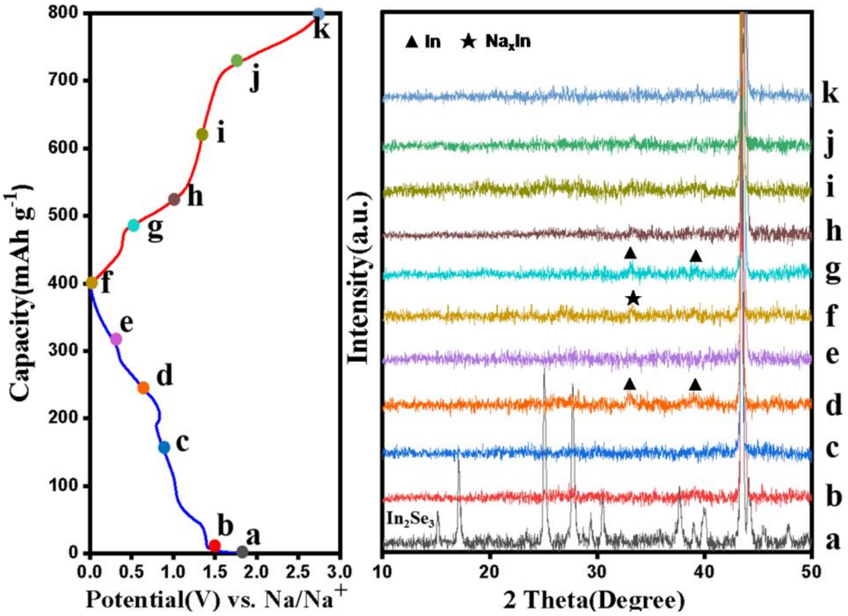

Figure S10 Ex-situ XRD patterns of the γ-In_2Se_3@rGO-0.06 electrode at different discharge/charge states.

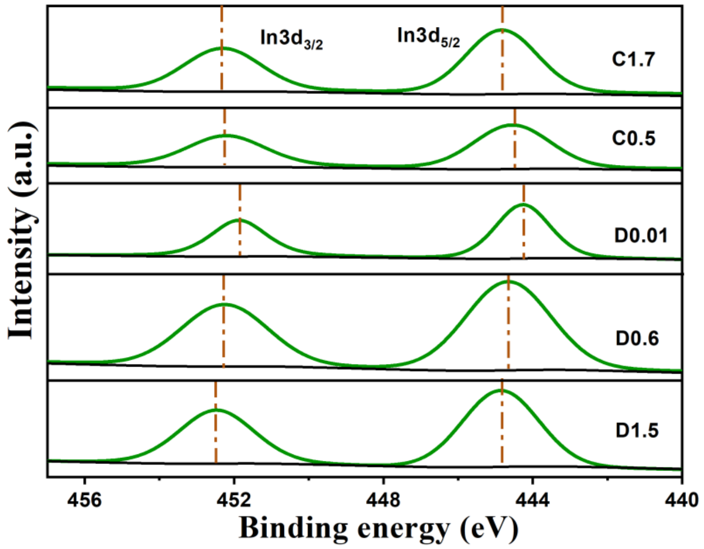

Figure S11 Ex-situ XPS spectra of the γ-In_2Se_3@rGO-0.06 electrode at different discharge/charge states.

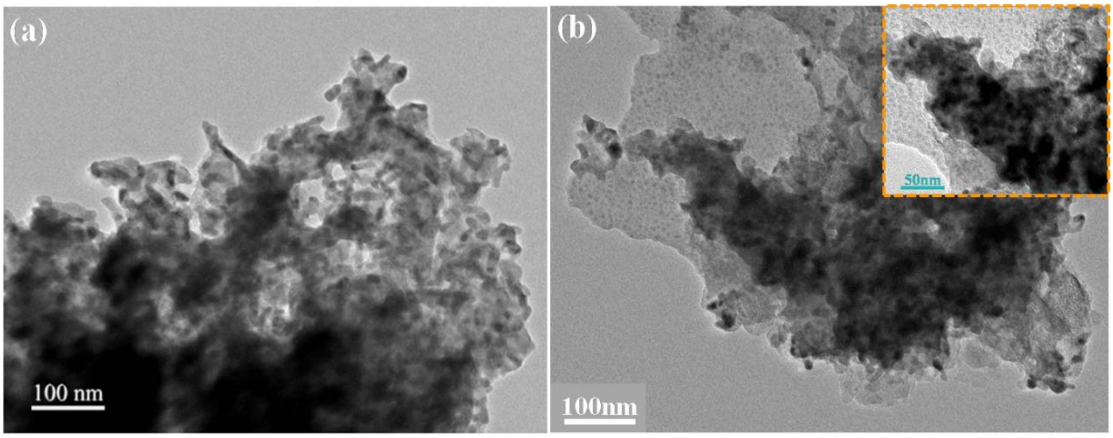

Figure S12 TEM images of (a) the bare In_2Se_3 electrode and (b) the γ-In_2Se_3@rGO-0.06 electrode after 10 discharge/charge cycles at 0.1 A g^{-1}.

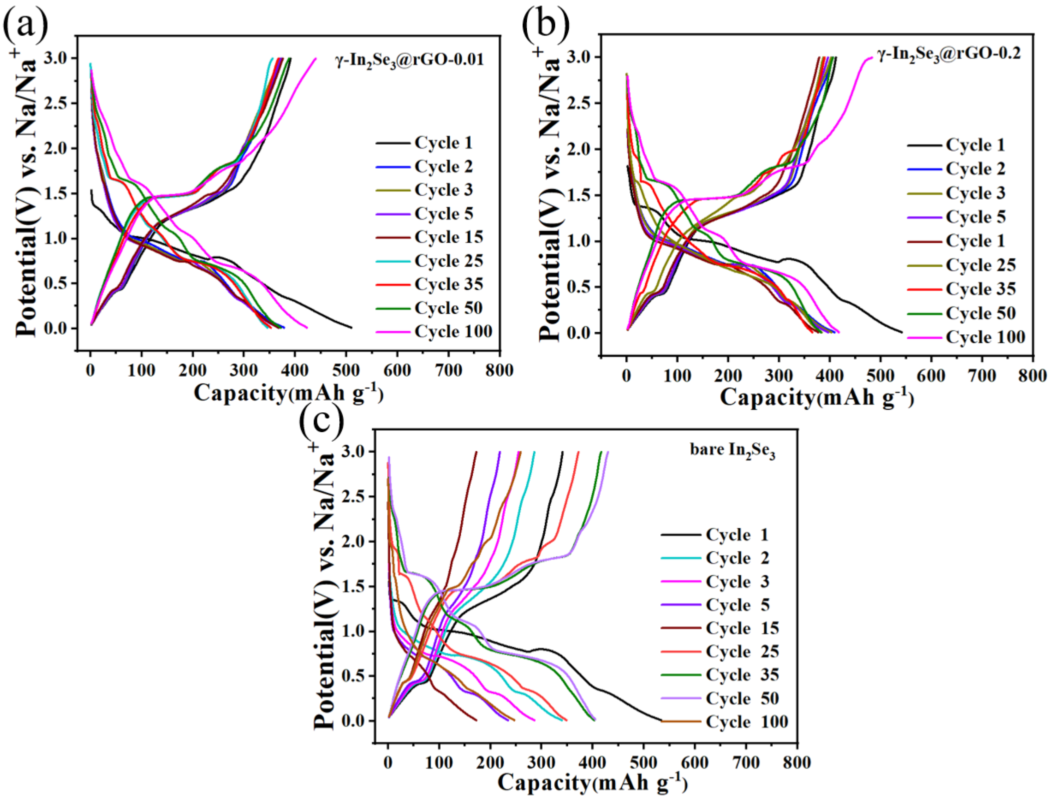

Figure S13 Discharge/charge curves of the γ-In_2Se_3@rGO-0.01, γ-In_2Se_3@rGO-0.2 and the bare In_2Se_3, respectively.

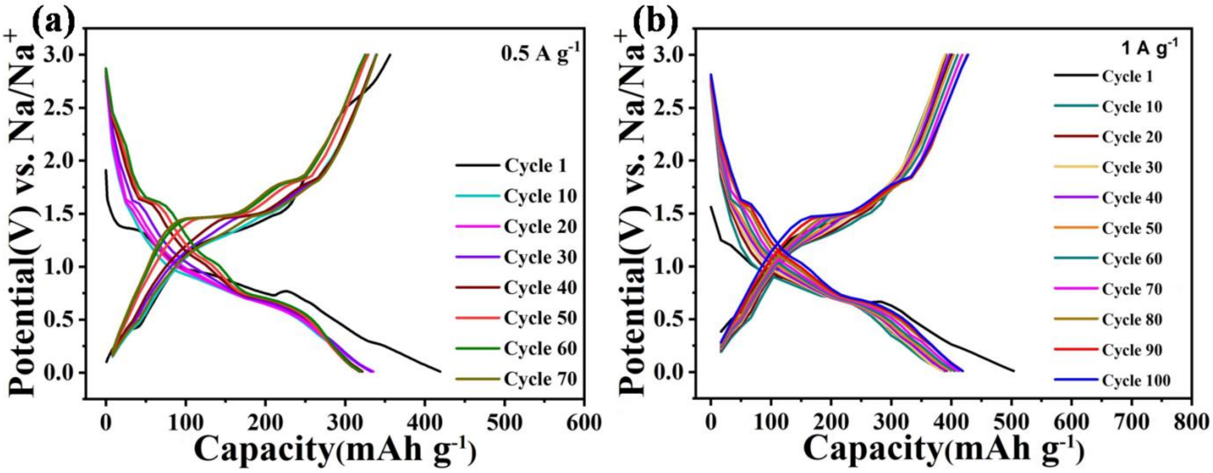

Figure S14 Discharge/charge curves of the γ-In_2Se_3@rGO-0.06 electrode at different current densities: (a) 0.5 A g^{-1}, (b) 1.0 A g^{-1}.

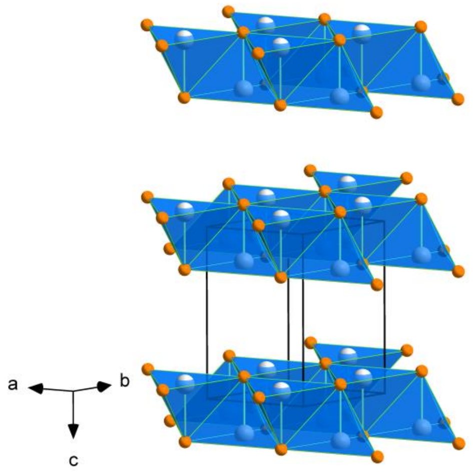

Figure S15 The typical layered structure of β-In_2Se_3 phase (JCPDS 34-1313, a=b=4.01 Å, c=9.64 Å).

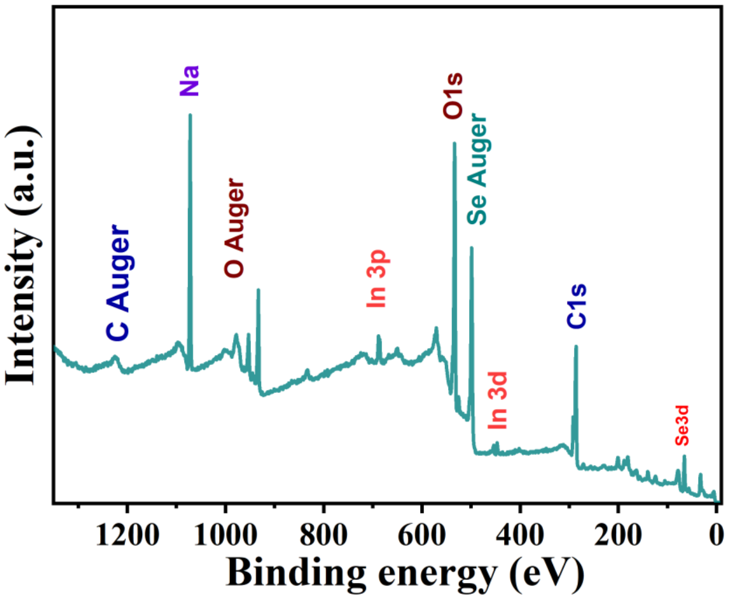

Figure S16 the XPS survey spectrum of the γ-In_2Se_3@rGO-0.06 nanocomposite after suffering 35 discharge/charge cycles.

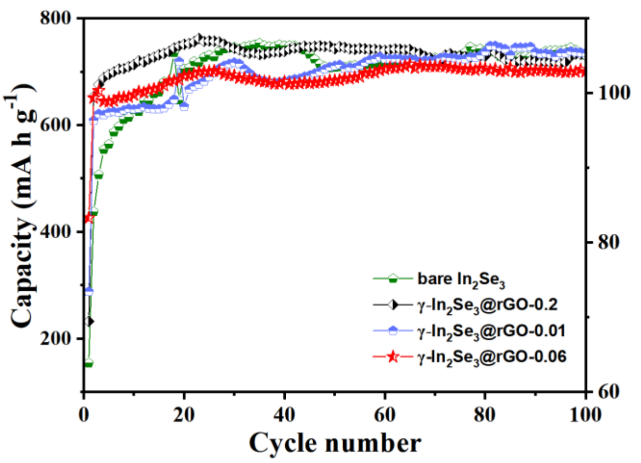

Figure S17 the Coulombic efficiencies of the In_2Se_3@rGO and bare In_2Se_3 electrodes with cycling.

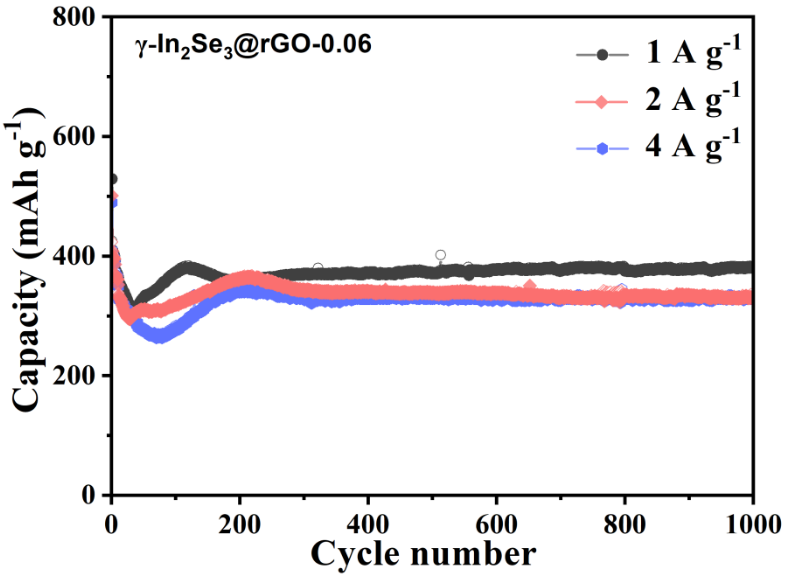

Figure S18 Long-term cycling stability of the γ-In_2Se_3@rGO-0.06 electrode at the current densities of 1.0, 2.0 and 4.0 A g^{-1}.

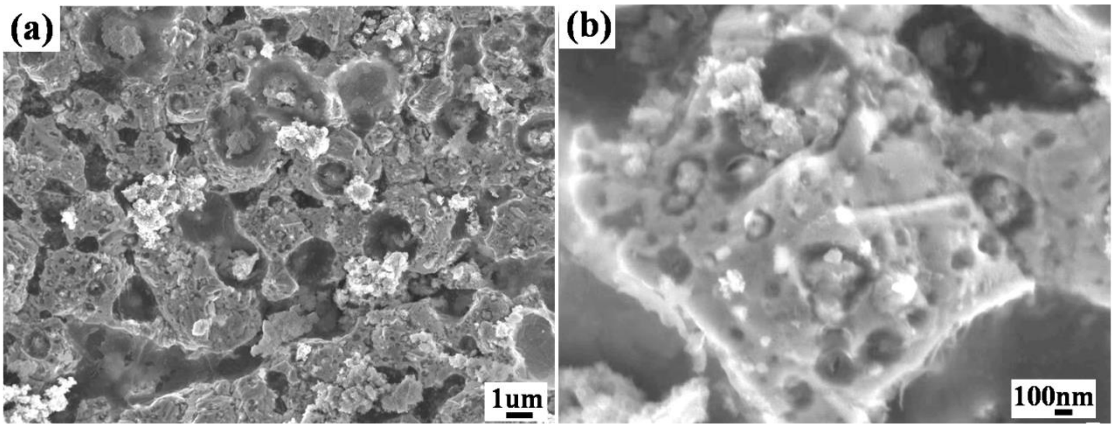

Figure S19 SEM images of the γ-In_2Se_3@rGO-0.06 composite after suffering 1000 discharge/charge cycles.

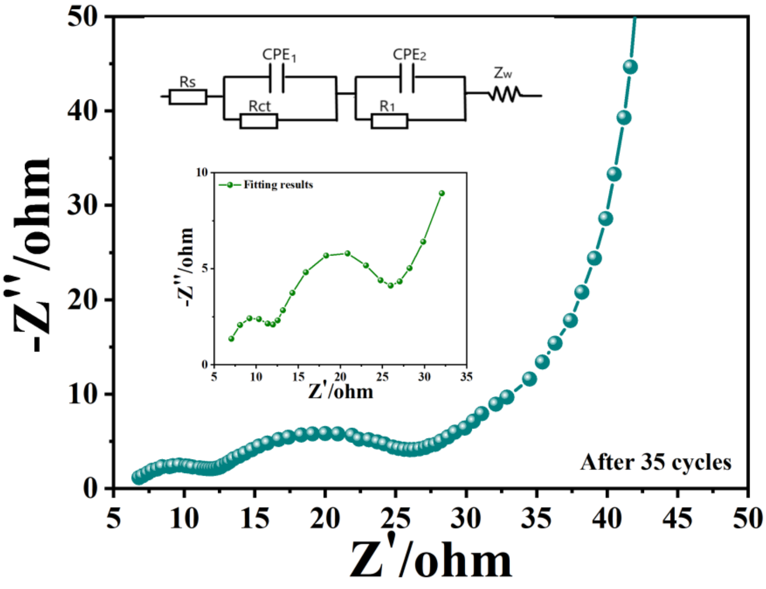

Figure S20 Nyquist plots of the γ-In_2Se_3@rGO-0.06 electrode after 35 discharge/charge cycles.

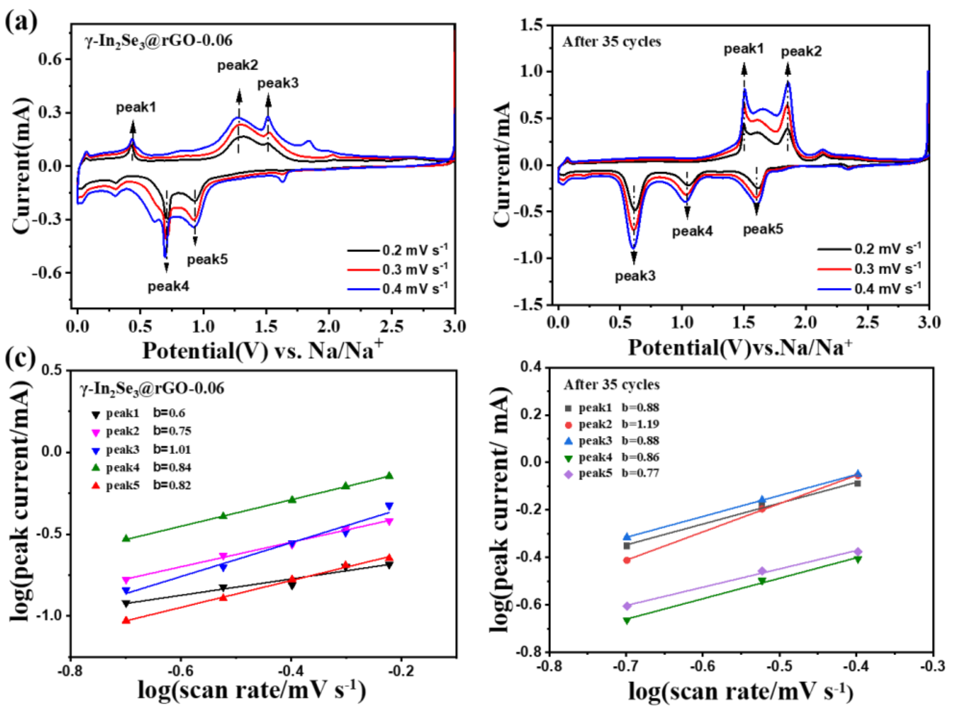

Figure S21 (a) and (b) are the CV curves at different scan rates for the γ-In_2Se_3@rGO-0.06 electrodes before and after 35 cycles, respectively. (c) and (d) are the corresponding b values of the redox peaks for the two electrodes.

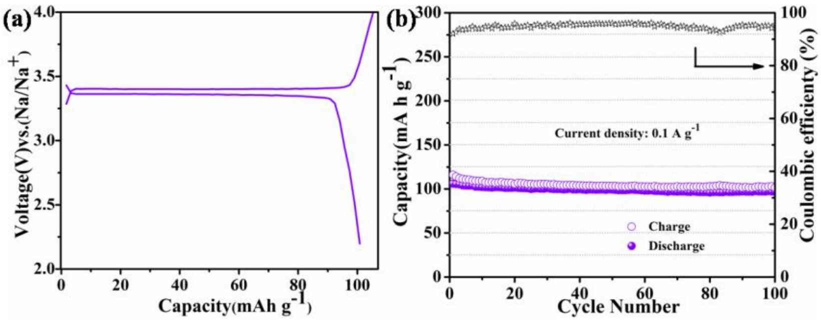

Figure S22 (a) Charge/discharge curve and (b) Cycling performance of the $Na_3V_2(PO_4)_3$ cathode in half-cell.

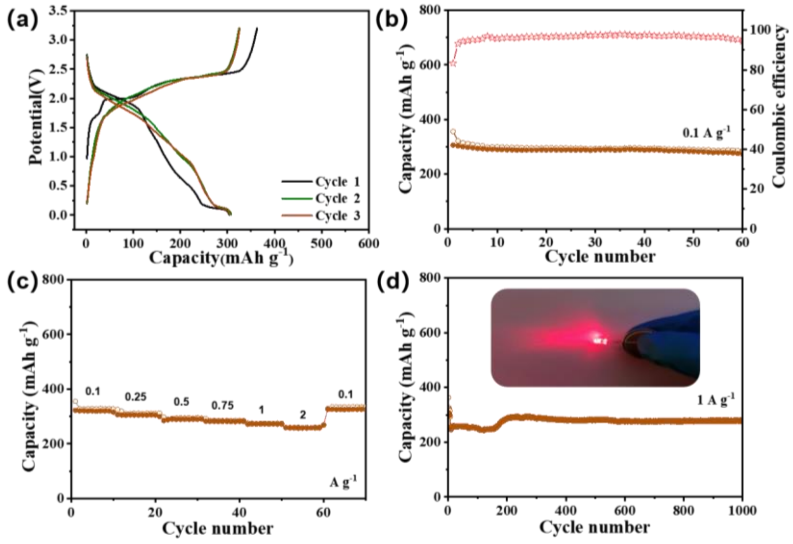

Figure S23 (a) The galvanostatitic charge/discharge profiles, (b) cycling performance, (c) Rate capability and (d) long-term cycling stability of the γ-In_2Se_3@rGO-0.06//$Na_3V_2(PO_4)_3$ full cell.

Table S1- Electric conductivity of the γ-In_2Se_3@rGO composites and bare In_2Se_3.

**Sample**	**Electric conductivity （S cm^{-1}）**
**Bare In_2Se_3**	0.010
**γ-In_2Se_3@rGO-0.01**	0.014
**γ-In_2Se_3@rGO-0.06**	0.050
**γ-In_2Se_3@rGO-0.2**	0.071

Table S2- The calculated proportions of rGO and PVP-derived pyrolysis carbon in the γ-In_2Se_3@rGO composites.

Sample	The proportion of rGO（wt.%）	The proportion of PVP-derived carbon (wt.%)
**γ-In_2Se_3@rGO-0.01**	3.15	0.25
**γ-In_2Se_3@rGO-0.06**	15.78	0.22
**γ-In_2Se_3@rGO-0.2**	45.36	0.14

Table S3- Performance comparison of the γ-In_2Se_3@rGO-0.06 electrode with the typical MS electrodes in SIBs reported recently.

Sample	Cycle performance (mAh g^{-1}) (A g^{-1}) (n)	Rate capaility (A g^{-1}) (mAh g^{-1})	Capacity retention ratio (%)	Ref.
**In_2Se_3@C/rGO**	393/1/500	5/322	69	32
**$Ni_{0.75}Zn_{0.25}$-SSe@C**	272/1/300	2/260	60	56
**$HCoSe_2$/ZnSe@NC**	265/5/800	10/245	50	57
**$CoSe_2$@NC/MWCNTs**	441/0.2/100	10/300	48	58
**$NiSe_2$@N-TCF/CNTs**	392/0.2/1000	2/334	78	59
**$Fe_7S_{8-x}Se_x$/CNTs**	314/2/1000	4/303	70	60
**$MoSe_2$@Mo_xN/C-I**	254/5/6000	10/235	44	61
**γ-In_2Se_3@rGO**	378/1/1000	20/272	75	This work

Table S4- Physical parameters of the γ-In_2Se_3@rGO electrodes after data fitting.

Sample	Rs(Ω)	Rct(Ω)	σ	Diffusion coefficients （$cm^2\ s^{-1}$）
**Bare In_2Se_3**	6.521	4.484	4.6	5.45×10^{-14}
**γ-In_2Se_3@rGO-0.01**	6.35	2.016	3.62	8.80×10^{-14}
**γ-In_2Se_3@rGO-0.2**	6.349	1.673	2.96	1.31×10^{-13}
**γ-In_2Se_3@rGO-0.06**	6.658	4.454	2.47	1.89×10^{-13}
**γ-In_2Se_3@rGO-0.06 After 35 cycles**	6.56	Rct+Rsf 4.68	3.11	1.19×10^{-13}
